# Mutant p53 achieved Gain-of-Function by promoting tumor growth and immune escape through PHLPP2/AKT/PD-L1 pathway

**DOI:** 10.7150/ijbs.67200

**Published:** 2022-03-14

**Authors:** Nannan Liu, Xinxiu Jiang, Leiming Guo, Chuchu Zhang, Meimei Jiang, Zhuoran Sun, Yizheng Zhang, Wunan Mi, Jiehan Li, Yang Fu, Feng Wang, Lingling Zhang, Yingjie Zhang

**Affiliations:** 1 School of Biomedical Sciences, Hunan University, Changsha, China.; 2 Department of Laboratory Medicine, the Third Xiangya Hospital, Central South University, Changsha, China.; 3 Department of Gastroenterology, Huadong Hospital, Shanghai Medical College, Fudan University, Shanghai, China.; 4 Department of R&D, Shanghai Creative Immune Therapeutics Co., Ltd, Shanghai, China.; 5 Department of Gastroenterology, the First Affiliated Hospital of Zhengzhou University, Zhengzhou, 450052, China.; 6 College of Biology, Hunan University, Changsha, China.

**Keywords:** mutant TP53, PHLPP2, AKT, PD-L1, immunity

## Abstract

The most frequent genetic alterations of the TP53 gene in human cancer were reported. TP53 mutation gains new function as a target of genetic instability, which is associated with increased tumor progression and poor survival rate in patients. In this study, more than three hundred colorectal cancer patients' samples were firstly analyzed, and the results showed that patients with mutant p53 had higher levels of AKT phosphorylation and PD-L1 expression, which were next verified both in cell lines *in vitro* and patients' samples *in vivo*. Further studies demonstrated that the hotspot of mutant p53 directly binds to the promoter of PHLPP2 to inhibit its transcription, and resulting in down-regulating its protein expressional level. Subsequently, AKT was released and activated, promoting tumor proliferation and metastasis. In parallel, 4EBP1/eIF4E was identified as downstream executors of AKT to enhance the translational level of PD-L1, which decreased the activation of T cells. Besides, inhibiting AKT/mTOR pathway significantly suppressed PD-L1 expression, tumor growth, and immune escape in p53 mutated cells. In conclusion, mutant p53 achieved its Gain-of-Function by transcriptionally inhibiting PHLPP2 and activating AKT, which suppresses immune response and advances tumor growth. Thus, this study provides an excellent basis for a further understanding of the clinical treatment of neoplastic diseases for patients with mutant p53, with an emphasis on immunotherapy.

## Introduction

The TP53 gene has a significant frequency of alteration in human cancers, which is associated with a high rate of tumor progression in an uncontrolled way. Li-Fraumeni syndrome has the possibility of a variety of early-onset cancers, which the underlying cause is those germline mutations 1. The mutant p53 can be divided into nonsense, frameshift, splice site, indel, missense, and other types 2. The reason is the structure of p53 protein folding dynamic change and intrinsically disordered domain 3, 4. Studies have shown that most p53 mutations have missense mutation preferentially occurred in the DNA binding domain, as a result of single amino acid change at many different positions 5. The TP53 “hotspot” mutant positions, including R175, G245, R248, R273, and R282 in the DNA binding domain, contribute to chemotherapy resistance and increase cell transformation, namely the Gain-of-Function 6. Missense mutation includes contact mutations (R273H) and conformational mutations (R175H) 7. For instance, the TP53R273H is associated with dysfunction or damage to the arginine's guanidinium group of DNA binding. Mutant p53 has been shown to play a vital role in all stages of tumorigenesis, including tumor initiation, promotion, and progression. However, little is known about the underlying mechanism of mutant p53 due to the complexity of p53 status and clinical treatment.

Previous studies have demonstrated that mutant p53 can induce the level of AKT and phospho-AKT related downstream signaling pathways. AKT is frequently also named Protein kinase B (PKB), a critical 57-kDa serine/threonine (Ser/Thr) kinase by phosphorylation. Phosphoinositide 3-kinase (PI3K) signaling pathway regulated AKT phosphorylation to suppress apoptosis. There are three mammalian isoforms (AKT1, AKT2, AKT3) of the AKT family, which play a central role in regulating cell survival, angiogenesis, and tumor formation, particularly the function of AKT1 8. AKT1 is phosphorylated by phosphatidylinositol-dependent kinase-1 at Thr308 and accompanied phosphorylation by mTORC2 complex at Ser473 after treatment with external stimuli during the process of cell signal transduction 9-11. It was indicated that phosphorylation and activation of AKT expression were remarked high in mutant p53 cells by western blotting in colorectal cancer, breast cancer, and endometrial cancer 12-14. It provides evidence that the TP53R273H mutation relies on the PI3K/AKT signaling pathway by suppressing the BCL2-modifying factor to promote cancer cell survival and tumor resistance. 13. Moreover, our former studies demonstrated that anti-proliferative activity and apoptosis were strictly dependent on SIRT6 activation through inhibiting AKT by BKM120 in colon cancer cells 15. Ipatasertib as a novel AKT inhibitor causes the transcription factor FoxO3a and NF-κB directly PUMA-dependent apoptosis 16. Our group also found that the anti-tumor efficiency of the small molecular AKT inhibitor SC66 through AKT/GSK-3β/Bax pathway 17. The above data suggested that AKT inhibitors worked both in wild-type p53 cells and in mutant p53 cells, which distinctly restrains cancer cell proliferation.

It was noticed that the PHLPP family (PHLPP1α, PHLPP1β, PHLPP2) could dephosphorylate the hydrophobic motif of specific AKT isozymes to negatively regulate its activity and to promote apoptosis 10, 18. Especially, PHLPP2 could dephosphorylate AKT1 and AKT3 at Ser473, while PHLPP1 could dephosphorylate AKT2 and AKT3 at Ser473 19. It leads us to think whether PHLPP2 could regulate cell progression because of the promoting effect of AKT on cell proliferation.

PI3K/AKT and the mammalian target of rapamycin (mTOR) signaling pathway inevitably plays a pivotal role in cancer progression 20-22. It commonly contributes to cell proliferation, survival, differentiation and drives the process of malignancy in patients with solid cancer 23, 24. It has been discussed the function of the PI3K/AKT/mTOR signaling pathway related to tumor mechanisms in many types of cancers. There are no extensive preclinical and clinical critical studies as yet having been reported.

Related studies have reflected that the mechanistic target of rapamycin and FK506-binding protein (mTOR) is a ubiquitously expressed serine/threonine (Ser/Thr) kinase. It is composed of two distinct complexes (mTOR1 and mTOR2), which regulated cell growth, proliferation, differentiation, and survival 25. The mTORC1 complex contains mTOR, regulatory-associated protein of mTOR (Raptor), mammalian lethal with SEC13 protein 8 (mLST8), 40-kDa proline-rich AKT substrate (PRAS10), and DEP domain-containing mTOR-interacting protein (DEPTOR), which is a master growth regulator that senses and integrates diverse nutritional and environmental cues, including growth factors, energy levels, cellular stress, and amino acids 26. The mTORC2 complex contains mTOR, Rictor, GβL, Sin1, PRR5/Protor-1, and DEPTOR, which promotes cellular survival by activating AKT, regulates cytoskeletal dynamics by activating PKCα, and controls ion transport and growth via SGK1 phosphorylation 27-29.

It is dispensable that mTORC1 signaling translational regulators were activated through eIF4E (eukaryotic translation initiation factor 4 epsilon) binding proteins 4EBP1, 4EBP2, and 4EBP3 (4EBPs) and ribosomal protein S6. The cap-dependent translation initiation factor eIF4E through phosphorylation at Thr-37 and Thr-46 of 4EBP1 to form an active initiation complex at the 5′ end of mRNAs as an oncogene 30-32. Moreover, the PI3K-AKT-mTOR oncogenic activity depends on the mTOR-4EBP1-eIF4E arm of AKT signaling, which is a major and specific controlling effector of protein synthesis.

Recently, it was reported that p53 signal and programmed death-ligand 1 (PD-L1) pathways play a crucial role in immunotherapy and the anti-tumor process 33, 34. Cortez et al. identified mutated p53 had low miR-34a and high PD-L1 levels compared to tumors with wild-type p53 in non-small cell lung cancer 35. The human PD-L1 gene is encoded by the CD274, located in chromosome 9p24.1. PD-1 binding receptor PD-L1, known as CD274 and B7 homolog 1 (B7-H1), is not only expressed in T cells, B cells, macrophages, and dendritic cells (DCs), but also existed in tumor cells and tissues 36-39. Immunosuppression has become a research hotspot in tumors to escape immune treatment 40. Furthermore, overexpressing PD-L1 will allow tumors to escape the effector-immune responses 38. Therefore, it is reasonable to study the critical function of the mutant p53 signaling pathway in cancer.

Although p53 mutation is a major drive of cancer, the incurable nature of cancer remains elusive. TP53 has a high mutation rate in colorectal cancer mainly due to the change of hydrogen bond interaction force. Mutant p53 has different structures compared with wild-type p53, which causes colon cancer cell progression and promotes tumor metastasis. Here, we showed that the PHLPP2-AKT pathway is a major target by which p53 promotes malignancy. In this research, we found p53 mutation could bind the promoter of PHLPP2 to down-regulate its expression, which induced AKT signaling pathway. Moreover, by analyzing the molecular mechanism of PD-L1 expression, the results showed a correlation between missense mutation of p53 and PD-L1. Mutant p53 was moreover essential for AKT-drive inducing PD-L1, in which 4EBP1 and eIF4E dependent translations were all integral.

## Materials and methods

### Cell lines and tissue treatment

The human colorectal cancer cell lines HCT116, HCT116^ p53-/-^, SW480, SW620, HT29, PANC-1, SKBR3, MC38, DC2.4, RAW264.7 were obtained from ATCC. The three cell lines (SW480, SW620 and HT29) were harboring a p53 missense mutation at codon 273 (p53R273H). The PANC-1 and SKBR3 cells were carried identical mutation profiles (p53R175H). These cells were grown in a medium of Gibco containing 10% heat-inactivated fetal bovine medium, which was cultured at 37 °C under a humidified 5% CO_2_ atmosphere in the incubator (Thermo Fisher Scientific).

### Colorectal cancer (CRC) Patient Samples

Surgical specimens from colorectal cancer patients were measured immediately using high throughput sequencing technologies in cancer genomics for study tumor-specific mutations in amino acid and therapy targets. Clinical specimens were frozen in liquid nitrogen for subsequent use immediately after excision. Next, the samples were cut into about green beans size to extract protein by western blotting. Put the other specimens into the sterile cryotubes, which can be tightly capped and been submerged in liquid nitrogen for other analysis.

### Data extraction and analysis

The colorectal cancer patients of RNA-Seq masked somatic mutation, clinical, and reverse-phase protein array (RPPA) data were downloaded from The Cancer Genome Atlas (TCGA-COAD dataset) and The Cancer Proteome Atlas (TCPA). The RNA-seq data were practicable in 449 TCGA-COAD patients, which have 223 subjects both in the RPPA data. The gene expression data were analyzed by the DESeq2 and EdgeR packages in R. GSEA analysis associated the gene signature with the TP53 missense mutant and TP53 wild-type patients using the R package GSEA. The Enrich-function-pathway dot-plots were evaluated by the ClusterProfiler package in R. The tumor immune microenvironment data were collected by R package xCell with gene expression.

### Structure determination

The information of the p53 protein sequence (PDB: 6GGC) was detected on the Protein Data Bank (PDB) database, which molecular three dimensional (3D) visualization by PyMOL software. The 3D structure of mutant p53 proteins after TP53 amino acid residues were mutated as p.R175H, p.R273H, p.R282W, or p.V274F. These mutant p53 protein hotspots were revealed in the DNA binding domain to affect its hydrogen bonding conformation.

### Plasmids and adenoviral vector

The RNAi (PLVX-shp53 and plko.1-shPHLPP2) systems were used to construct stable cell lines with knocked down genes (p53 and PHLPP2) by an RNAi method. Non-targeting control shRNAs (p53 and PHLPP2) were cloned into vectors (PLVX-shRNA2-Puro and plko.1). The p53R273H cDNA was cloned into the lentivirus vector pcDNA3.1-3xFlag-T2A-EGFP. The mCherry-PHLPP2 cDNA was cloned into the lentivirus vector PLVX-mCherry-N1. The shAKT vector (pYr-3.1-shAKT) and negative control (pYr-3.1) were designed and produced.

Lentiviruses expressing shRNA knocked down was generated by transfecting 1.5 μg of ps-Pax2, 0.5 μg of pCMV-VSVG, and 2 μg of plasmid using lipofectamine 2000 in 293T cells. The culture viral supernatant was harvested 48 and 72 hours post-transfection. Mix polybrene (5 μg/mL) with viral particle. Infected cells were selected with puromycin or hygromycin for 3 days after 72 hours in the completed medium.

### Western blotting

The collected cells were lysed on ice for 30 min using RIPA lysis buffer (Beyotime Institute of Biotechnology) supplemented with proteinase inhibitor and phosphatase inhibitor (Selleck). The protein concentration was measured by bicinchoninic acid (BCA) protein assay kit (Thermo Fischer Scientific). The 30μg protein of samples was contained in SDS-PAGE. Then the proteins were transferred into PVDF membranes before blocked with 5% no-fat milk for 1 hour at room temperature. The PVDF membranes were incubated in antibodies' solution at 4 °C overnight. Next, the membranes were washed three times with PBST for ten minutes. This was followed by incubated in the secondary antibodies for 1 hour and to rinsing three times in PBST. It was detected by an enhanced chemiluminescent kit (New Cell & Molecular Biotech) using Odyssey infrared imaging system (LICOR, Lincoln, NE).

### Chromatin immunoprecipitation

The ChIP assays were performed using a CHIP assay kit (Beyotime Institute of Biotechnology) following provided reagents and protocol. The colorectal cells were cross-linked with formaldehyde for 10 min, which was terminated with glycine solution for 5 min at room temperature. Then the cells were incubated on ice before lysed in SDS lysis buffer. The genomic DNA was sonicated in an ultrasonic breaker machine to obtain 200-1000 bp DNA fragments. The cell lysates were added NaCl at 65 °C for four hours to remove cross-links between protein and genomic DNA. The immunoprecipitations were probed with antibodies against p53 (Proteintech) or normal rabbit IgG (Invitrogen) overnight at 4 °C and protein A+G Agarose/Salmon Sperm DNA. Subsequently, we washed the DNA-protein complex three times at 4 °C. The ChIP products for PCR detection after DNA purified to use the universal DNA purification kit (Tiangen). The sequence primers of the PHLPP2 of the predicted four sites used in this study were presented in Table 2.

### Real-time quantitative PCR

With the RNAsimple Total Kit (Tiangen), total RNA from cancer cells was collected in RZ buffer. The RNA quality and quantity were measured with NanoDrop ND-1000. Besides, the RNA integrity was assessed by standard denaturing agarose electrophoresis. The synthesis of 500 ng of total RNA via reverse transcription using High Capacity cDNA Reverse Transcription Kit (Thermo Fisher) produced cDNA. The RT-qPCR assay was performed by using ChamQ^TM^ Universal SYBR qPCR Master Mix (Vazyme). Gene-specific primer sequences are listed in Table 2.

### Immune flow cytometry

The shp53, Pifithrin-α (PFTα) HBr, and PQC-p53R172H overexpressing in MC38 cells were transfected with lipofectamine 2000 for 48 hours. The MC38 cells supernatant were collected before broken by ultrasonic waves, which were injected in DC2.4 cells medium to co-cultivate for 18 hours. These cells incubated in different conditions were collected, washed by PBS with 0.5% BSA. Then it resuspended by 100 μL PBS with 0.5% BSA and incubated with 1 μL of fluorescent antibody for 30 min in dark at 4 °C. Next 200 μL PBS with 0.5% BSA was added to each sample and immediately performed flow cytometric analysis by the number of staining cells. Data generated using BD FACS Calibur and analyzed using the FlowJo-V10 software.

### Immunohistochemistry

Surgically resected colorectal samples were fixed in 4% buffered paraformaldehyde solution overnight. Then place these tissues in 15% sucrose gradient solution and 30% sucrose in PBS at room temperature overnight. The collected tumors were embedded in optimal cutting temperature compound, frozen to -80 °C fridge, and sliced into 5 μm using a cryostat microtome, which was placed into warm water with a pair of tweezers before using glass slides to remove tissues in an incubator at least 2 hours. The tumor sections were permeabilized with 1% TritonX-100 after were washed three times with PBS. The samples were blocked at room temperature for 45 min, which incubated overnight at 4 °C with primary antibodies of PD-L1, p53, AKT, Ki67, CD4, CD8. The sections were washed three times, incubated with the relevant secondary antibodies for 1 hour at room temperature. The tumor sections were washed 3 times with PBS, stained with DAPI, and mount with Fluoromount G. The images were capture and analyzed by Olympus IX73 microscope.

### Statistical analysis

The data plotted graphically by using the GraphPad Prism version 6.02 software and the R version. To compare the expression value of PHLPP2 and PD-L1, quantitative statistics were presented as mean ±standard deviation (SD). The P-values of AKT, TP53, PD-L1, and macrophages were calculated with the long-rank test. In addition, we used the Kaplan-Meier log-rank test to compare the survival curves of patients. The differences between groups were tested for the T test and two way ANOVA. All statistical tests were two-sided and the value of P<0.05 was considered statistically significant.

## Results

### Mutant p53 promotes malignant proliferation of colon cancer

**Figure 1:**
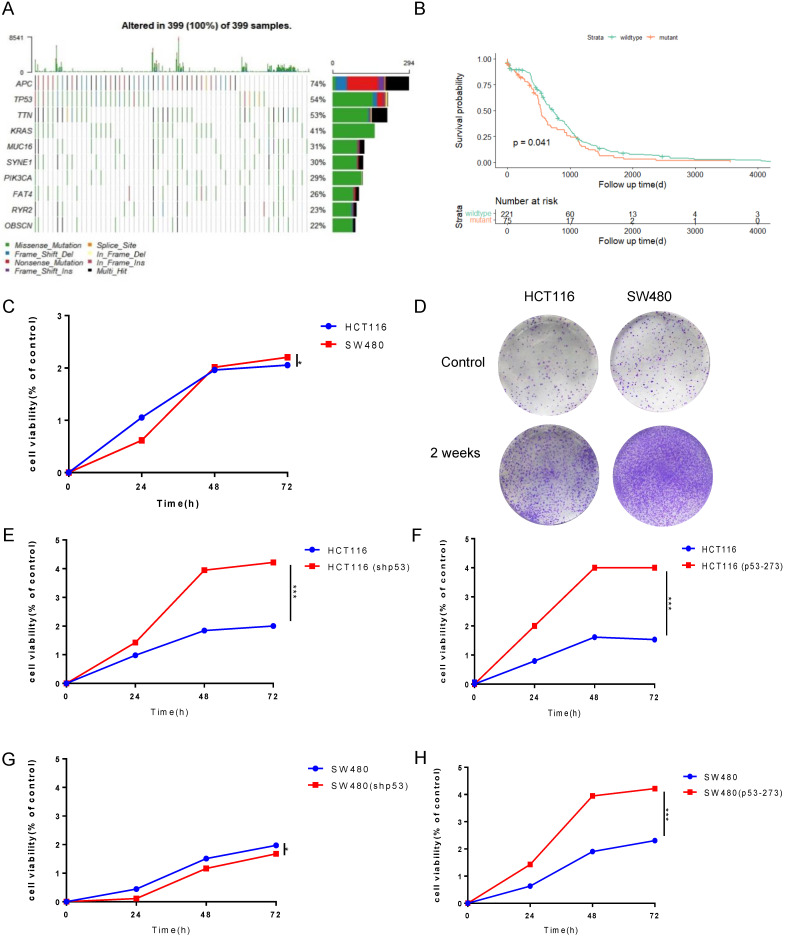
** Mutant p53 promotes the malignant proliferation of colon cancer. (A)** The prevalence has susceptible gene mutations in 399 patients of colorectal cancer. **(B)** With a follow-up period of 4000 days, the Kaplan-Meier survival curves of colorectal cancer patients with wild-type p53 significantly higher than missense mutant p53 (log-rank). **(C-H)** The data obtained to use cell counting kit-8 assays revealed that p53R273H (F, H) and shp53 (E, G) had a significant effect on the proliferation of wild-type p53 cells (HCT116) under normal condition.

P53 missense mutation profile data of colorectal cancer was collected through the R-maftools package from The Cancer Genome Atlas (TCGA-COAD dataset). The data from oncoplot figure was used to analyze the mutational landscape of 399 samples by clinical features (**Figure 1A**). This picture showed a variety of mutant forms of COAD samples characterized by the accumulation of genetic alterations, which include missense mutation, frame shift deletion, nonsense mutation, and so on. It indicates that the proportion of p53 mutation rate was up to 54%, which was the second on the list. Therefore, considering p53 was an important prognostic factor, we divided these patients into p53 missense mutant and wild-type groups to perform a survival analysis. Patients, who were detected p53 missense mutation, had a lower survival rate than patients with wild-type p53 (P=0.041). Based on the results above, p53 missense mutation was considered as a prognostic characterization of patients in **Figure 1B**.

To explore the higher oncogenic potential caused by mutant p53R273H in colorectal cancer cells, CCK8 assay and colony formation analysis were performed and we found that the cell-based viability of SW480 was higher than the performance of HCT116 in this study (**Figure 1C, 1D**). Next, to further confirm the function of WT/Mutant p53 in proliferation, wild-type p53 was firstly knocked down in HCT116 cells using shRNA, and showed a more rapid growth (**Figure 1E**), which was expected, as WT p53 is a tumor suppressor. By the contrast, mutant p53R273H was overexpressed in HCT116 cells in the complementary experiments, which accelerated cell growth (**Figure 1F**), meaning mutant p53 is a tumor promoter. Mutant p53R273H was also knocked down in SW480 cells, which obviously slow down cell proliferation (**Figure 1G**). Identical complementary experiments were performed in SW480 cells and similar results were obtained (**Figure 1H**). These results indicated that mutant p53 promotes cancer cell progression.

To study the structure-function aspects of genetic variants in Silico analysis and verification, we have investigated the potential effects of the structure of variant p53 protein. The picture showed no significant difference between wild-type and mutant p53 in three-dimensional structures (**Supplementary Figure 1A, 1B**).

There are well noticed that the DNA binding sequence of amino acids linked with the conformational switch of p53 crystal structures. Through protein-ligand interaction analysis, hydrogen bonds were observed on the Asp-184, Pro-191, Gln-192, His-179 in wild-type TP53R175H (**Supplementary Figure 1C**), the Leu-133, Cys-135, Asp-281 in wild-type TP53R273H (**Supplementary Figure 1E**), the Thr-125, Tyr-126, Glu-286 in wild-type TP53R282W (**Supplementary Figure 1G**) and the Ser-240 in wild-type TP53V274F (**Supplementary Figure 1I**). It predicted that the p.R175H variant lost three hydrogen bonds except for Gln-192 (**Supplementary Figure 1D**) and the p.R273H variant lost one hydrogen bond except for Met-133 and Cys-135 (**Supplementary Figure 1F**). Similarly, we found the p.R282W variant lost three hydrogen bonds Glu-286 in **Supplementary Figure 1H**. However, it was noticed that the p.V274F variant has not changed in **Supplementary Figure 1J**. Our results support that hydrogen bonds are critical in the structure and function of both WT and mutant p53 proteins.

### Mutant p53 activates AKT to promote tumor growth

**Figure 2:**
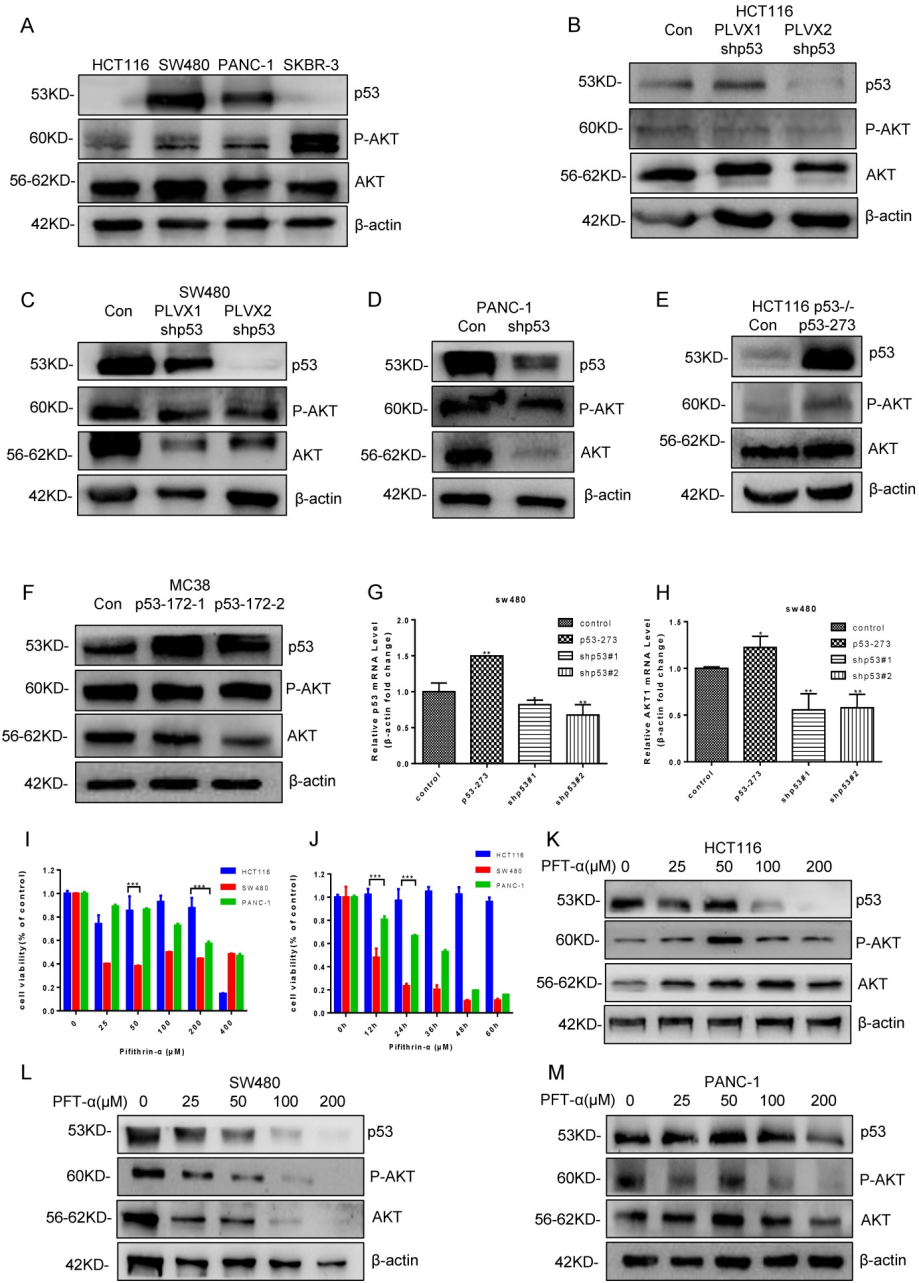
** Mutant p53 activates AKT to promote tumor growth. (A)** The effect of AKT phosphorylation was higher in SW480, PANC-1, and SKBR-3 cells compared with HCT116 cells under normal conditions. **(B-D)** The expressing shRNA for p53 lysed and lysates from HCT116 (B), SW480 (C), PANC-1 (D) cells blotted with anti-P-AKT and anti-AKT antibodies. **(E-F)** The lysates of HCT116 ^p53-/-^ (E) and MC38 (F) cells overexpressing vector or p53R273H subjected to western blotting with indicated antibodies. **(G, H)** The qPCR assay was utilized to measure p53 and AKT1 mRNA level in SW480 cells. **(I, J)** Cell viability was measured by using CCK-8 post-treatment with pifithrin (PFT)-α at 0, 25, 50, 100, 200, and 400 µM after 24 hours or 0, 24, 36, 48, 60, and 72 hours, respectively. **(K, L, M)** The protein levels of P-AKT and AKT were determined by western blotting in the vehicle control or pifithrin (PFT)-α treated HCT116 (K), SW480 (L), and PANC-1 (M) cells.

To study AKT activation status in p53 wild-type and mutant cells, the phosphorylation level of AKT was detected, and we found P-AKT level increased significantly in p53 mutant cells (SW480, PANC-1, SKBR3), which promoted the occurrence and proliferation of cancer cells (**Figure 2A, Supplementary Figure 2A**). After knockdown of p53 with siRNA, the level of P-AKT reduced markedly in various human cancer cells, including colon cancer cells HCT116, SW480 (p53R273H) and pancreatic cancer cells PANC-1 (p53R175H) (**Figure 2B-2D, Supplementary Figure 2B-2D**). By the contrast, overexpressing mutant p53 restored the P-AKT level in HCT116 p53^-/-^ cells (**Figure 2E, Supplementary Figure 2E**). Interestingly, the expression level of total AKT also decreased evidently after knocking down mutant p53R273H in SW480 and PANC-1 cells, which increased after overexpressing mutant p53R273H in HCT116 p53-/- cells (**Figure 2C-E**). However, this phenomenon did not happen in mouse colon cancer cells (MC38) with over expressing mutant p53R172H, which might be attributed to the differences between human and mouse cells or mutation sites (**Figure 2F, Supplementary Figure 2F**). To further study the mechanism of mutant p53 regulating AKT expression, RT-PCR was performed to quantify the AKT mRNA levels after over expressing or knocking down mutant p53R273H in SW480 cells, which gave us identical trends as that of the AKT protein levels (**Figure 2G-2H**). These results indicated that mutant p53 could also up-regulate AKT expression through transcriptional level, which could be controlled in a PHLPP2-independent manner.

Pifithrin-α (PFTα) HBr is an effective inhibitor of p53, which inhibits the p53 dependent transcription. To find a proper dose in our system, HCT116, and SW480 cells were treated with various doses of PFT-α. Cell viability was analyzed by using Cell Counting Kit-8 after PFT-α stimulation for 24 hours (**Figure 2I**). As a result, HCT116 cell viability was hardly inhibited by PFT-α, while a significant inhibiting effect was observed in SW480 cells. Consistently, 100 uM PFTα was used for further investigating and cell viability at different time points (0, 24, 36, 48, 60, and 72 hours) after stimulation was tested, which gave us similar results (**Figure 2J**).

Furtherly, the level of P-AKT was measured in HCT116, SW480 and PANC-1 cells, which were treated with a variety of doses of PFT-α. As expected, PFT-α decreased the expression of both wild type and mutant p53 in those cells. However, the level of P-AKT had opposite trends. It had a little bit increase in HCT116 cells, but decreased gradually and significantly in SW480 and PANC-1 cells (**Figure 2K-2M, Supplementary Figure 2K-2M**). Therefore, PFT-α appears to inhibit P-AKT in a mutant p53 dependent manner.

Although we have reported that mutant p53 causes chemotherapy resistance due to loss of function of inactivating PUMA transcription 41, it is suggested that we should also explore gain of function with mutant p53. Previous studies add to these results suggesting that AKT inhibitors have the function to suppressed cell proliferation in mutant p53 cancer cells. Thereafter, it can further manifest mutant p53 gain of function in lymphoma cells, as p53 and AKT inhibition by GNE477, SC66 and PFT-α suppressed tumor growth (**Supplementary Figure 3A-3J**).

### Mutant p53 activates AKT through directly binding to PHLPP2 promoter and down-regulates its transcription

**Figure 3:**
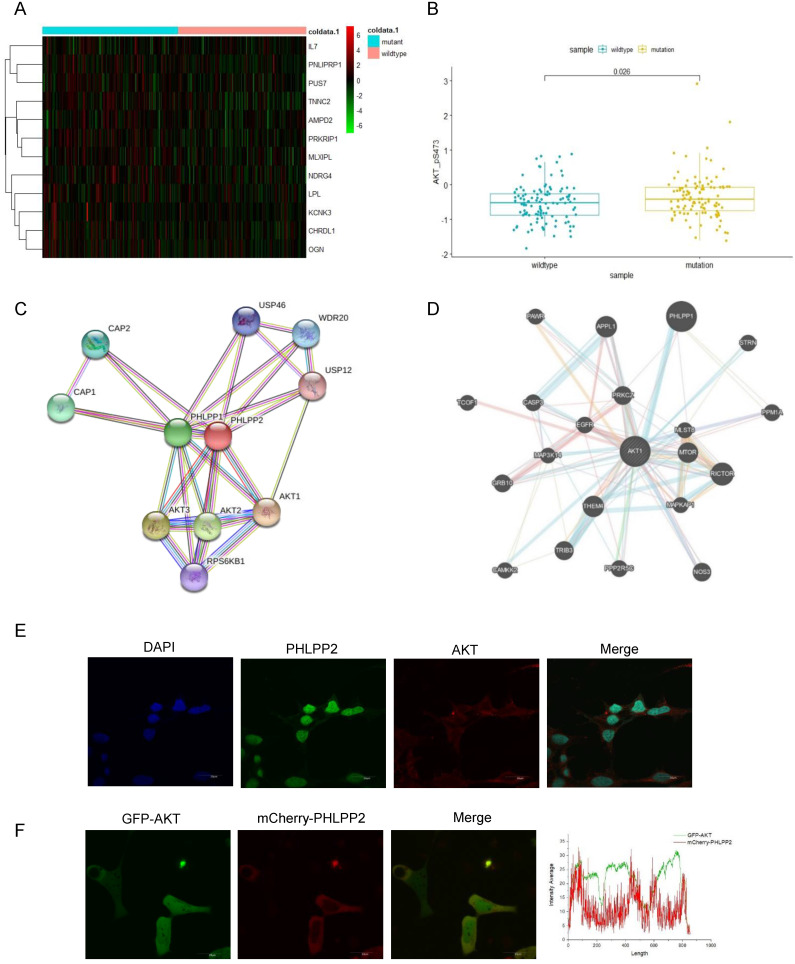
** Mutant p53 activates AKT through directly binding to the PHLPP2 promoter and down-regulates its transcription. (A)** Heatmap representation of relative mRNA expression of AKT-mTOR pathway showed significant hotzone in p53 missense mutation group but not in wild-type colorectal patients. **(B)** Correlation analysis of P-AKT mRNA levels in wild-type and mutant p53 colorectal cancer patients from TCGA, p=0.026. **(C, D)** The BioGRID and GeneMANIA databases provide comprehensive datasets of PHLPP2 interactions for the protein encoded by AKT. **(E)** The co-localization of PHLPP2 with AKT was observed by immunohistochemistry in HCT116. **(F)** The live HCT116 cells expressing GFP-AKT were transfected with mCherry-PHLPP2 for 48h, thereafter the cells were imaged by confocal microscopy.

To investigate whether mutant p53 activated AKT to promote tumor development, we generated a heat-map to illuminate the differences of AKT-mTOR signaling pathway between p53 mutant and wild-type groups (**Figure 3A**). We also analyzed AKT phosphorylation level between the p53 mutant and wild-type groups in TCGA, which showed an induced AKT activation in the p53 mutant group of colon cancer, p=0.026 (**Figure 3B**). The related mRNA level of P-AKT was highly enriched with mutant groups. Next, the interacting proteins with AKT were further evaluated, and a certain number of potential candidates were predicated by BioGRID and GeneMANIA databases (**Figure 3C-3D**), which indicated that AKT activation might be dependent on specific PHLPP isoforms. To gain insight into how PHLPP2 inactivated and dephosphorylated AKT, we respectively observed the consistent presence of two proteins by fluorescent imaging in a single cell. The immunofluorescence detection was utilized to visualize the localization chemically labeled with PHLPP2 and AKT antibodies under the microscope (**Figure 3E**). Moreover, the colocalization of mCherry-PHLPP2 and GFP-AKT was screened in the same cells after plasmid transfection (**Figure 3F**).

**Figure 4:**
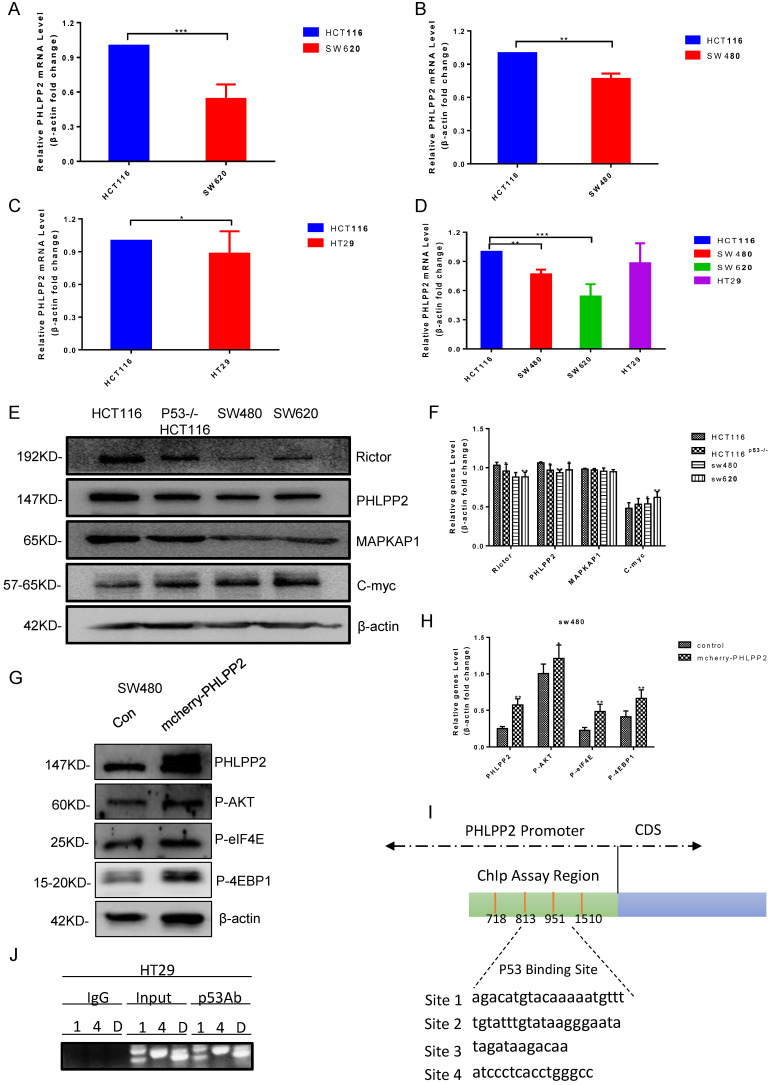
** PHLPP2 was found to interact with AKT. (A-D)** The logarithmic scale 2-ΔΔCt was used to calculate the relative PHLPP2 expression in HCT116, SW480, SW620, and HT29 cells. **(E-H)** The expression PHLPP2 was analyzed by western blotting analysis in colorectal cells. **(I, J)** Schematic representation of the human PHLPP2 promoter region, which predicted four p53 binding sites.

To study the relation between PHLPP2 and mutant p53 in human cancer cells, RT-qPCR and western blot were performed to analyze PHLPP2 mRNA and protein levels in HCT116, SW620, SW480, and HT29 cells. It was observed that both mRNA and protein levels of PHLPP2 were significantly lower in colorectal cancer cells with mutant p53 compared to those with wild-type p53 (**Figure 4A-4F**). Moreover, to explore the exact role of PHLPP2, we detected the decreased activity of phospho-AKT, which has been overexpressed PHLPP2 in SW480. It suggested that PHLPP2 inhibited the activation of AKT. We extend our findings to increase the PHLPP2 protein expression by transfection method for studying the function of regulated molecules signaling, such as 4EBP1 and eIF4E. The western blotting assay showed that PHLPP2 was efficiently increased after transfection of mCherry-PHLPP2. It also indicated that the level of phospho-AKT, 4EBP1, and eIF4E increased along with the expression of PHLPP2 (**Figure 4G-4H**). Consistently, to assess whether mutant p53 can directly regulate PHLPP2 in cancer cells, we firstly presume that mutant p53 may bind to the promoter of PHLPP2. Then, the hTFtarget database website was used to predict the binding conditions and four potential sites were obtained (**Figure 4I**). Finally, the activity of the different regions on PHLPP2 promoter was detected, followed with ChIP assay. The results proofed that mutant p53 has a direct binding with PHLPP2 promoter in the predicted region (**Figure 4J**). These results above indicated that mutant p53 directly inhibited PHLPP2 transcription, which leaded to the activation of AKT.

### Mutant p53 represses the immune response level in cancer

**Figure 5:**
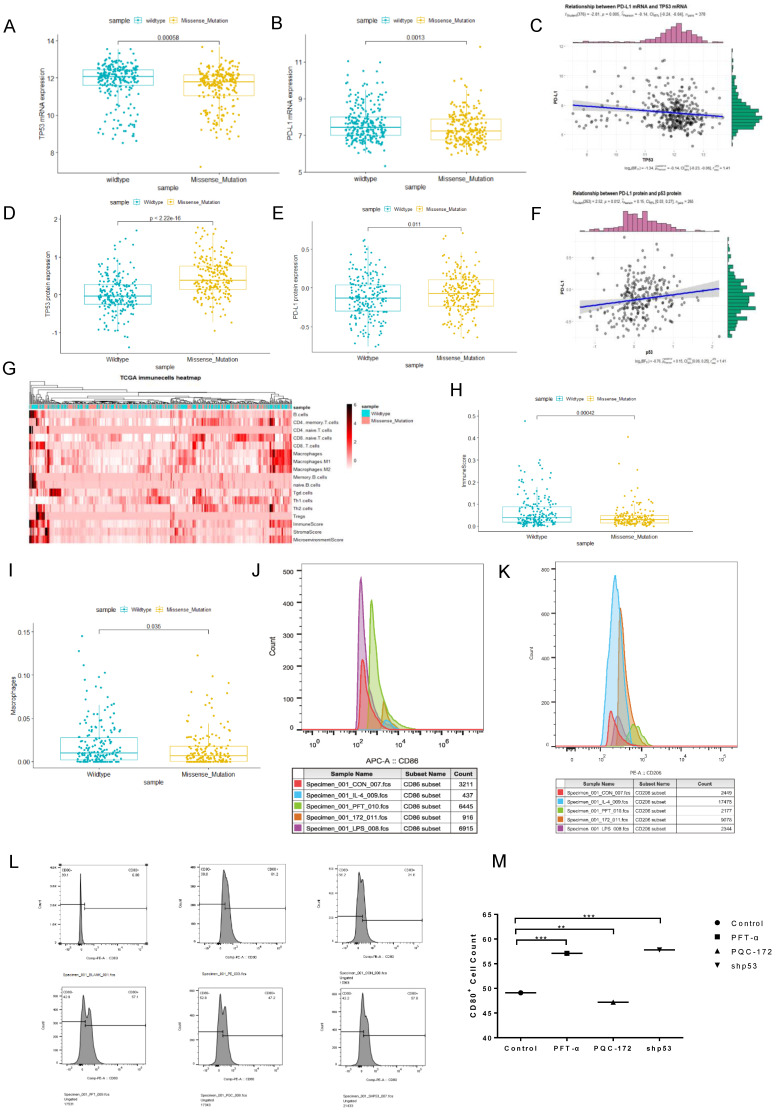
** Mutant p53 represses the immune response level in cancer. (A, B, D, E)** Correlation between PD-L1 (CD274) and status of p53 mRNA and protein level in samples of colorectal data from TCGA and TCPA databases. **(C, F)** Correlations between p53 status and PD-L1 (CD274) levels in colorectal patients p53 missense mutation and p53 wild-type based on analysis of the TCGA and TCPA databases. **(G)** A heatmap is plotted to describe the key components of missense mutant p53 and wild-type p53 groups of the tumor microenvironment (TME) in colorectal cancer. **(H)** Clinical data from TCGA were to study immune score among missense mutant p53 and wild-type p53 groups. **(I)** Clinical data from TCGA were to study macrophage infiltrations among missense mutant p53 and wild-type p53 groups. **(J, K)** RAW264.7 cells were treated with pifithrin-α (PFTα) HBr (200 µM), PQC-p53R172H, LPS (100 ng/ml), IL-4 (20 ng/ml) for 24h. It was harvested and stained with fluorescent antibodies against CD11b, F4/80, CD86, and CD206 and analyzed by flow cytometry. **(L, M)** For the co-culture condition, DC2.4 cells were given 18 hours before being transfected with shRNAs for p53 or overexpressed plasmid of PQC-p53 and treated with pifithrin (PFT)-α in MC38 cells for the same time, flow cytometry revealed the number of CD80+ cells.

Studies have shown that tumor microenvironment (TME) was significant to the relationship between tumor growth and progress 42, 43. We downloaded RNA sequencing (449 samples) and protein profile data (223 samples) of the studied colon cancer type in the TCGA and the Cancer Proteome Atlas (TCPA) databases. After analyzing, we found the p53 mutant group (p=0.00058) showed decrease in p53 mRNA level compared with that in p53 wild-type group (**Figure 5A**), which had an opposite trend for the protein level (**Figure 5D**) (p<2.22e-16). Similar trends were observed for PD-L1 in patients with mutant or wild-type p53 (**Figure 5B,** p=0.0013 **and 5E,** p=0.011). Furthermore, a positive correlation on protein level but not mRNA level between p53 and PD-L1 was identified (**Figure 5C and 5F**).

Tumor-associated macrophages (TAMs) are supposedly played a critical role in tumor cell invasion, motility, and intravasation while stimulating angiogenesis, suppressing the immune response, and preventing tumor cell attack by natural killers, and T cells 44. It is well known that M2 macrophages or neutrophils level is served as a negative prognostic marker. The heat-map was intended to describe key compositions of TME in colon cancer (**Figure 5G**). Additionally, the quantity of CD4+ memory T-cells was less in missense mutant p53 samples compared with that in wild-type p53 samples. The crucial role of Perp, a p53 pro-apoptotic target, in mediating the persistence of CD4+ effector memory T-cell undergoing lymphopenia-induced proliferation 45. It showed that CD4 memory resting T cells and M1 macrophages may be correlated with the TMRS (tumor metabolic risk score), which corresponded to immunoediting in p53 mutant lung adenocarcinoma patients 46. Antigen-specific CD4(+) T cell proliferation was related to the down-modulation of p53 47. Trp53R172H/+ mouse pancreatic ductal epithelial tumor promotes neutrophil recruitment and down-regulates CD3+ T cells, CD8+ T cells, and CD4+ T helper 1 cells to resistant immune therapy 48. And our results showed that the immunescore in patients with wild-type p53 appeared higher compared with that in patients with mutant p53, p=0.00042 (**Figure 5H**). Expectedly, the quantity of macrophage was also less in p53 missense mutant samples, p=0.035 (**Figure 5I**).

Macrophages and dendritic cells were essential members of the innate immune system. We focus on the role of mutant p53 in shaping the immune functions of macrophages and dendritic cells. In the co-culture of macrophage cells with mouse cancer cells experiments, the M0 macrophage cells (RAW264.7) were activated by different stimulatory cytokine cocktails to derive M1 macrophages and M2 macrophages. After being exposed to MC38 cell lysates that were treated with different stimulations, it was detected by Flow cytometry. The occupancy markers of M0 macrophages (RAW264.7), CD11b and F4/80, were found decreased after co-cultured with MC38 cell lysates in which over expressed PQC-p53R172H. When compared with M1 and M2 macrophages, effects of LPS and pifithrin-α (PFTα) HBr were observed in activated M1 macrophages, while effects of IL-4 and PQC-p53R172H were observed in activated M2 macrophages (**Figure 5J and 5K**).

To investigate the role of mutant p53 in immune response in tumor, DC2.4 cells were co-cultured with MC38 cell lysates that were treated in three different ways. The result showed that the quantity of CD80^+^ cells decreased after overexpressing mutant p53 (PQC-p53R172H), however, increased after knocking down p53 or treating with PFT-α (**Figure 5L-5M**). The results of Figure 5J-5M suggested that mutant p53 represses the response level of immune cells to cancer cells.

Further analysis confirmed this view point by showing the PD-L1 mRNA level, progression-free survival curves and other immune indexes of colorectal patients in different therapeutic groups, including radandphar (9 samples), normal, notreat (181 samples), pharmaceutical (48 samples) and radiation (46 samples). The patients of the radanaphar group had the highest PD-L1 mRNA level while showed the worst survival probability. By the contrast, the patients of the radiation group had the lowest PD-L1 mRNA level while showed the best survival probability (**Supplementary Figure 4A and 4B**). The results indicated that there is a negative correlation between PD-L1 expression level and survival curves.

Next, each group was divided into p53 missense mutant and wild-type subgroups to compare the immune phenotypes between them. As a result, the quantity of CD4^+^ memory T cells, CD8^+^ naïve cells, and macrophages was lower in the p53 mutant subgroups for the radio and pharmaceutical therapies (**Supplementary Figure 4C and 4D**). And the quantity of B cells, CD4^+^ memory T cells and macrophages was also lower in the p53 mutant subgroups for the untreated patients (**Supplementary Figure 4E and 4F**). Over all, mutant p53 reduced the immune score of the colorectal patients and **Supplementary Figure 4G** indicated that radio and pharmaceutical therapies dropped the immunocompetence.

### Mutant p53 up-regulates PD-L1/CD274 via AKT pathway

**Figure 6:**
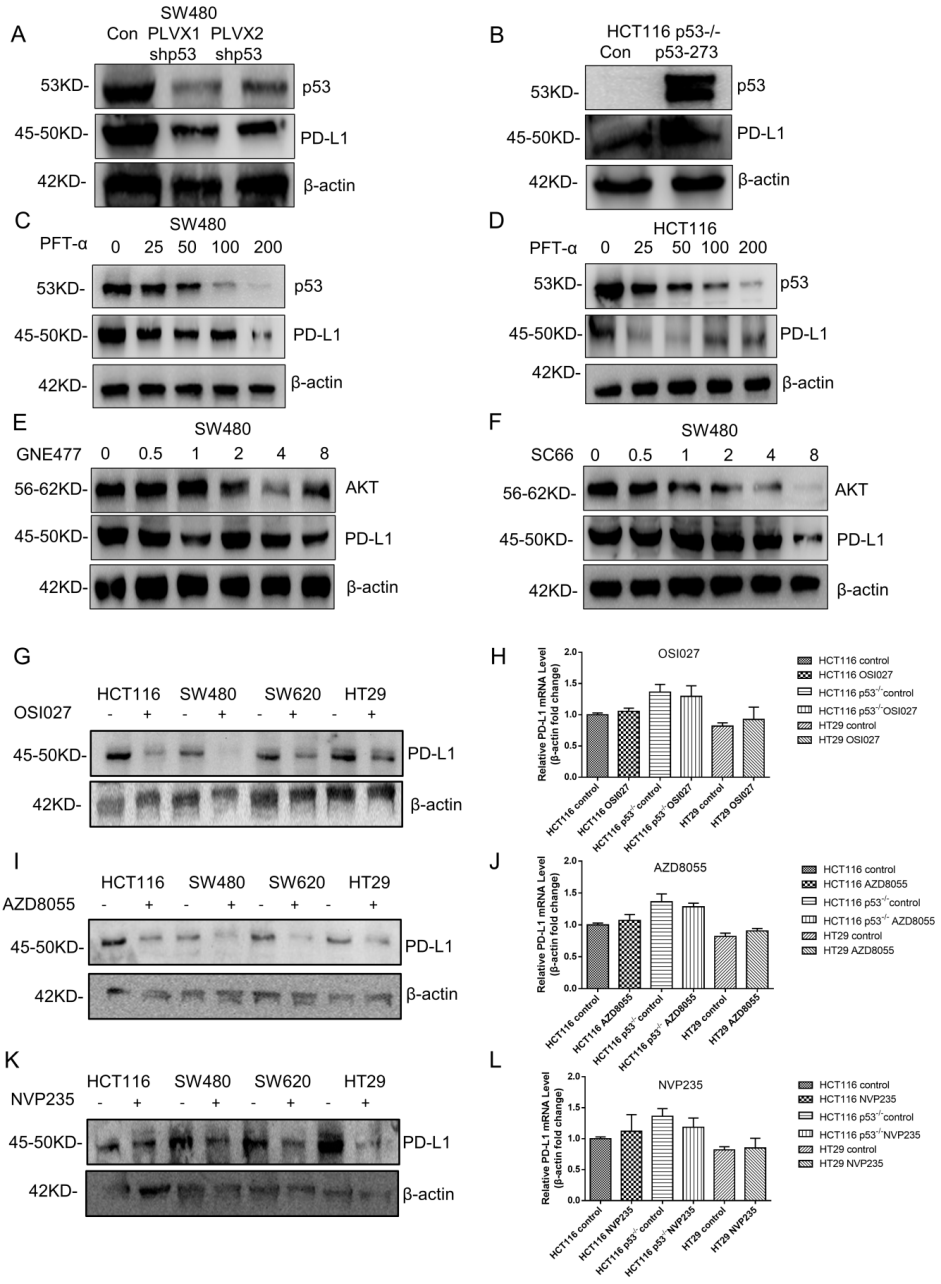
** Mutant p53 up-regulates PD-L1/CD274 via the AKT pathway. (A)** SW480 cells were transfected with stably expressing control or shRNAs for p53 before harvest. **(B)** Lysate from HCT116 p53^-/-^ cells expressing shRNAs for p53R273H immunoblotted with the indicated antibodies. **(C, D)** The colorectal cells (SW480, HCT116) were treated with pifithrin (PFT)-α for 24 hours. **(E, F)** Lysates were prepared from SW480 cells treated with GNE477 or SC66 for 24 hours and analyzed by immunoblotting with the anti-AKT and anti-PD-L1 antibodies. **(G, I, K)** The expression of PD-L1 was detected by western blotting in four colorectal cells after mTOR inhibitors for 24 hours. **(H, J, L)** Quantitative real-time PCR detected relative PD-L1 (CD274) expression in human colorectal cells. The logarithmic scale 2ΔΔCt was used to calculate the relative PD-L1 (CD274) expression treated with inhibitors OSI027 (H), AZD8055 (J), NVP235 (L).

Currently, little is known about the molecular mechanism of PD-L1 expression in colorectal cancer, which was investigated in this study. The result showed that the PD-L1 expression level declined after knocking down mutant p53 (**Figure 6A, Supplementary Figure 5A**), while increased when mutant p53 was over expressed (**Figure 6B, Supplementary Figure 5B**). Besides, PFT-α treatment significantly decreased the expression level of PD-L1 in SW480 cells (**Figure 6C, Supplementary Figure 5C**) and HCT116 cells (**Figure 6D, Supplementary Figure 5D**).

As the downstream mediator and executor of mutant p53, the function of AKT was also evaluated.

The PI3K/mTOR inhibitor GNE477 and AKT inhibitor SC66 were used to suppress AKT activity in SW480 cells, which caused the obvious decrease on PD-L1 expression level (**Figure 6E and 6F, Supplementary Figure 5E, 5F**). Furthermore, other PI3K/mTOR inhibitors AZD8055, NVP-BEZ235 and OSI027 were also recruited to treat SW620 and HT29 cells, which gave us the similar results (**Figure 6G, I and K, Supplementary Figure 5G-5I**). However, for the mRNA level, the comparison among p53 WT, knockout and mutant cells after treatment has no big significance (Figure 6H, J and L, **Supplementary Figure 5J**), which was consistent with the results of patients' sample analysis. Therefore, these results indicated that mutant p53 up-regulated PD-L1 expression through activating AKT, and presented a possibility on the study of PD-L1 translational regulation in the current medical treatment.

### AKT/4EBP1/eIF4E enhances PD-L1 translational level in colon cancer with mutant p53

**Figure 7:**
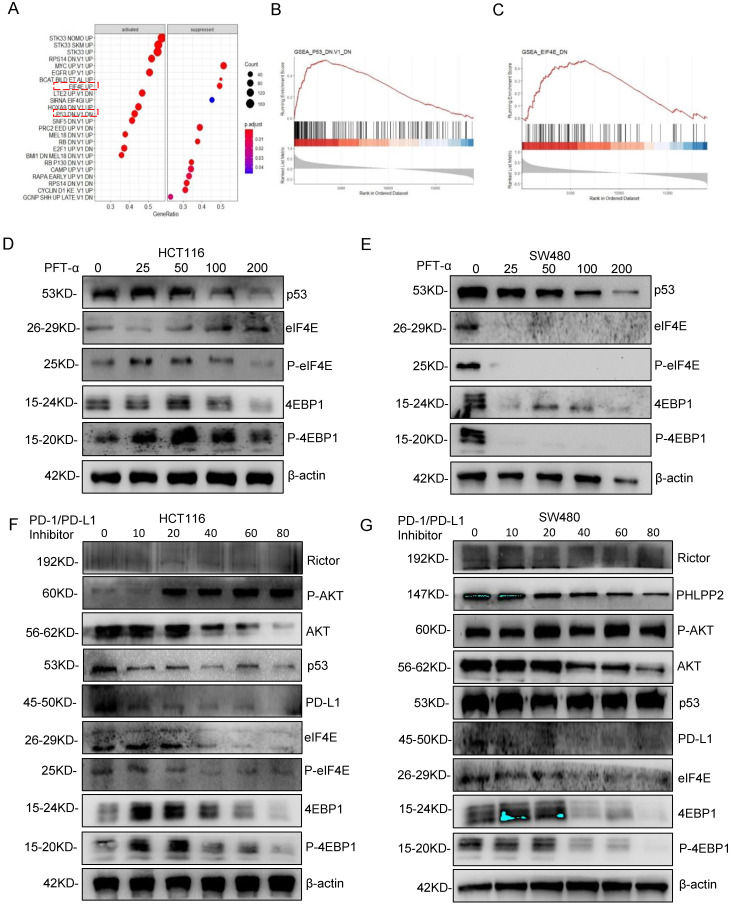
** AKT/4EBP1/eIF4E enhances PD-L1 translational level in colon cancer with mutant p53. (A)** The results of gene set enrichment analysis (GSEA) showed that PD-L1 significantly regulated with the p53 signaling pathway and eIF4E pathway. **(B, C)** Regarding the enrichment plot p53 signaling pathway and eIF4E pathway. **(D, E)** The HCT116 and SW480 cells were treated with pifithrin (PFT)-α at 0, 25, 50, 100, 200, and 400 µM after 24 hours. Lysates from these cells were immunoblotted by the indicated antibodies. **(F, G)** The HCT116 (F) and SW480 (G) cells were treated with PD-1/PD-L1 inhibitor for 24 hours at different doses between 0 and 80, respectively. Lysates from these cells were immunoblotted with indicated antibodies and β-actin was used as a loading control in western blotting analysis.

To further explore the mechanism of PD-L1 regulation, GSEA analysis was performed based on RNA sequence data from the TCGA database. The results showed a prominent enrichment of p53 (p=0.425) and eIF4E (p=0.5) signaling pathways (**Figure 7A-7C**), which revealed that the activation of p53 and eIF4E is critical for PD-L1 up-regulation.

Next, the predicted results were verified by western blotting detection. As shown in **Figure 7D, 7E, Supplementary Figure 6A and 6B**, phospho-eIF4E and phospho-4EBP1 levels decreased significantly in SW480 cells after mutantp53 inhibition by PFT-α, which was not observed in HCT116 cells. Interestingly, total eIF4E and 4EBP1 also declined markedly by induced by PFT-α treatment, which did not happen in HCT116 cells either, indicating that the activation of 4EBP1/eIF4E depends on mutant but not WT p53. To further confirm it, mutant p53 was knocked down or overexpressed, and the expression level of eIF4E, 4EBP1 and PD-L1 was detected, which gave us similar results (**Supplementary Figure 7E, 7F**). In addition, the phospho-eIF4E and phospho-4EBP1 decreased with administration of the PD-1/PD-L1 inhibitor 1 (**Figure 7F, 7G**) and BMS-1001 (**Supplementary Figure 7C, 7D**), which exhibited a potent feedback inhibitory effect. To explore the differences of PD-L1 related signaling pathways between the mutant p53 and wild-type p53 samples, GSEA was utilized to analyze RNA sequence data from TCGA, which revealed a prominent enrichment in interferon-gamma signaling (**Supplementary Figure 7A-7B**).

### Mutant p53 increases tumor growth and PD-L1 expression in colorectal cancer patients

**Figure 8:**
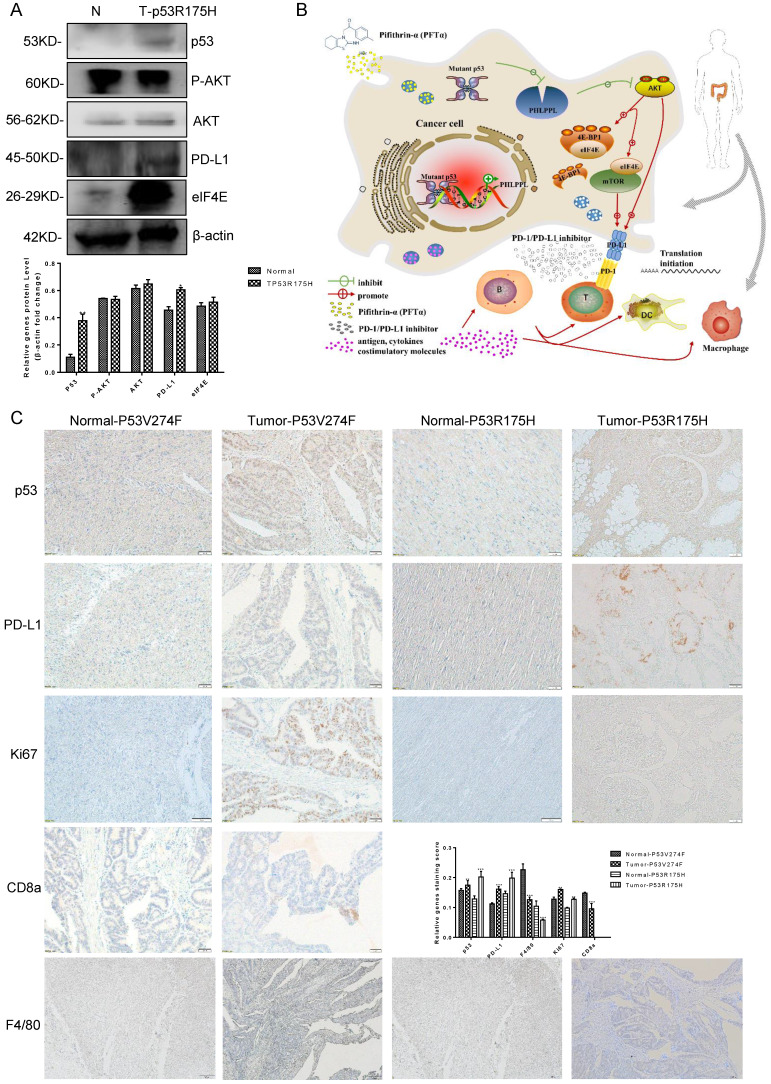
** Mutant p53 increases tumor growth and PD-L1 expression in colorectal cancer patients. (A)** To study the expression of p53 and PD-L1 or AKT in patients, lysates from tissues by immunoblotted with indicated antibodies were analyzed in colorectal cancer patients. **(B)** Schematic representation of missense mutant p53 related immune therapy signaling pathway. **(C)** Representative images depicting immunohistochemical (IHC) staining of p53, PD-L1, Ki67, CD8a, and F4/80 of p53 missense mutation and wild-type in colorectal tissues.

13 confirmed colorectal cancer cases underwent surgical resection and the samples were harvested, then the high throughput sequencing technology was recruited to analyze the mutant status of p53 (**Table 1**). The results showed that 7 patients' samples of 13 have mutant p53, including R175H, R282W, V274F and V173L. As shown in **Figure 8A**, high protein expression levels of p53 and PD-L1were observed in p53 mutant samples compared with those of the normal tissues. IHC assays were performed and similar results were obtained. Furthermore, the proliferation marker Ki67 revealed strong signal specifically surrounding the mutant p53 tissues. Besides, CD8a and F4/80+ macrophages were detected in a section of p53 mutant colorectal cancer tissue (**Figure 8C**). The statistical analysis supports the mechanism of p53 missense mutation regulates immune checkpoint. Collectively, all of these data demonstrated that mutant p53 transcriptionally inhibits PHLPP2 and activates AKT/4EBP1/eIF4E pathway to up-regulates PD-L1 expression, subsequently suppresses the immune response and executes its Gain-of-Function in tumor growth (**Figure 8B**).

## Discussion

Previous studies have shown that mutations in the TP53 gene frequently lead to the p53 protein with oncogenic property and Gain-of-Function. Nonetheless, researchers have failed to coalesce around biological or molecular mechanisms concerning different properties of specific p53 mutation. Indeed, a recent analysis showed that some p53 mutations could not predict efficacy in lung adenocarcinomas (LUAD) patients treated with immune checkpoint inhibitors (ICIs). It suggested that p53 missense and nonsense mutations were associated with PD-L1 expression, IFN-g signatures, and TME composition, which were remarkably divergent as ICIs biomarker in terms of p53 mutation heterogeneity 49. PI3Kα-selective inhibitor BYL719 (Alpelisib) could downregulate mutant p53 with MYC-dependent expression, which is treated with Head and neck squamous cell carcinoma (HNSCC) 50. It has been shown that K-ras^G12V^ activation accelerated ROS generation and induced FGFR1 expression, resulting in a significant upregulation of PD-L1 51. While mutant p53 executed its gain-of-function to drive tumor progression by exerting more intracellular ROS 52. Another study also shows ROS level is higher in p53 mutant pancreatic ductal adenocarcinoma (PDAC), which lead to the accumulation of miR-135 53. Here, we identified the action of missense mutant p53 associated immune signaling network that could be combined inhibitors used in colorectal cancer.

Several major domains of the mutant p53 tetramer are similar with that of wild-type p53. While the major mutation of p53 is localized in the DNA binding domain, protein function is determined by its complex structure. The polar interaction networks of the spatial structure were changed in variant protein, especially in hydrogen bonding interaction, which has great importance for the structure and function of the protein (**Supplementary Figure 1C-1J**). The different chains were highlighted with different colors. Specifically, the Arginine located in the sites, Arg175 and Arg273, in wild-type p53 is transformed into Histidine in mutant p53, respectively, which was marked in red.

Previous studies have reported that AKT could phosphorylate MDM2 at Ser166 and Ser186 sites to negatively regulate wild-type p53 levels in the PI3K/AKT signaling pathway 54. A recent study also showed that MDM2 inhibitor ALRN-6924 could activate p53 to induce endogenous retroviruses (ERVs), providing effective immunotherapy 55. The latest report found that FBXO31 could inhibit cervical cancer progression through PI3K/AKT-mediated MDM2/p53 axis 56. In a recent study, mutant p53 had been shown to exert its oncogenic expression function through microRNAs and many transcriptional modulators 57-59. Besides, some studies have suggested that AKT is a potential therapy target via Rac1 signaling contributes to the Gain-of-Function of mutant p53 14, 60. Nevertheless, the molecular mechanism of mutant p53 mediated AKT activation cross-talk nodes among wild-type p53 is still poorly understood. Results from this study showed that mutant p53 significantly through phosphorylated AKT to activate its expression in colorectal, breast, pancreatic cancer (**Figure 2**). We found that mutant p53 activating AKT in two way, by down-regulating PHLPP2 expression to enhance AKT phosphorylation level, or by transcriptionally up-regulating AKT expression level.

Studies have clarified that ENH (Enigma Homolog Protein) promotes the phosphorylation of AKT by PHLPP2 61. Furthermore, PHLPP2 could inhibit cell viability by dephosphorylating AKT 18. Studies based on the samples of patients with metastatic prostate cancer proved that co-deletion of PTEN and PHLPP1 was tightly correlated with TP53 and PHLPP2 62. When the combination of PTEN and p53 was deleted in the mouse of prostate epithelia, I16 could activate PHLPP2 to drive the expression of myc, which promoted cell proliferation 63. We have found that mutant p53 R273H down-regulated the expression level of mRNA and protein of PHLPP2 in colorectal cancer (**Figure 4A-4F**). Mechanistically, mutant p53 could bind to the promoter of PHLPP2 (**Figure 4I, 4J**). When performing analysis by confocal fluorescence microscopy, PHLPP2 was determined that it could interact with AKT by different spectral variants of the green fluorescence protein (GFP) and mCherry red fluorescent protein (mCherry) in **Figure 3F**. PHLPP2 could be an interesting anti-cancer target in the context of immunochemotherapy, further *in vivo* studies are needed to confirm whether PHLPP2 inhibition/activation could affect the therapeutic efficacy of immunotherapy in mutant or wild-type p53-driven cancers.

It is well established that mutant p53 promotes tumor progression and increases oncogenic transformation through gain of function mechanism. There has been plenty of recent studies focusing on the observation that p53 played an important role in immune escape of tumor. Mutant p53 interacts with TBK1 to inhibit the cGAS/STING cytoplasmic DNA pathway, which weakens type I interferon response by immune escape to promote tumor progress 64. To explore the mechanism of mutant p53 contributing to tumor progression through immune escape, we attempted to identify the markers of immunity by bioinformatics methods, which collected abundant colorectal samples from the TCGA database (**Figure 5A-5I**). We demonstrated that PD-L1 expression was regulated at the translational level by AKT. Besides, some studies have illustrated this point. The eEF2K regulates PD-L1 expression at the translational level of its mRNA by virtue of a uORF in its 5'-region 65. IFN-γ and LPS provoked PD-L1 combined with p85 of phosphatidyl inositol 3-kinase (PI3K) to activate AKT signaling in neutrophils 66. It showed that the AKT-mTOR pathway was initiated into PD-L1 expression with IFN-γ in human LUAD and squamous cell carcinomas 67. In there, mutant p53 expression in colorectal cancer was mostly regulated by PD-L1 through the AKT signaling pathway at the translation level (**Figure 5A-5F**). Tumor associated macrophages (TAMs) behavior was influenced by mutant p53 forms. Mutant p53 could repurpose TAMs to support tumor development (**Figure 5I**). After the activation of T cell, the MHC-I peptide recognized the T cell reporter and then upregulated PD-1, which kept the immune system in check. Cancer cells could inhibit T cell activation by overexpressing PD-L1.

Clinical studies have shown that targeting PD-1/PD-L1 is related to tumor immune evasion, however, the role of p53 in regulating PD-L1 remains elusive. It revealed that wild-type p53 decreases PD-L1 by up-regulating miR-34, which is directly bound to the PD-L1 3' untranslated region in non-small cell lung cancer, and HCT116 cells without p53 have higher PD-L1 expression 35, 68. It was also observed that IFN-γ induced PD-L1 expression upon p53 knock down, which correlated with JAK-STAT signaling pathway 68. While what would happen if wild-type p53 was replaced by mutant p53? Some studies have shown that there is some relationship between mutant p53 and PD-L1 69, 70. However, the molecular mechanism behind is unclear. In our present study, we found that mutant p53 up-regulated PD-L1 expression markedly, which through the PHLPP2/AKT pathway. It is interesting that wild-type p53 decreased PD-L1 expression, which was restored by knocking out wild-type p53. While over expressing mutant p53 in p53-/- cells further increased PD-L1 expression, indicating that different states of p53 have different influences on PD-L1. Our study demonstrated that p53 mutation exhibited an increased PD-L1 protein level than wild-type p53 (**Figure 5E**). The expression of PD-L1 was estimated according to preceding a variety of treatments by using the log-rank test. PFT-α could decrease PD-L1 expression in mutant p53 cells (**Figure 6C, Supplementary Figure 5C**). Furthermore, AKT was associated with PD-L1 expression in the missense mutant p53 group (**Figure 6E, 6F, Supplementary Figure 5E, 5F**). In groups of cells underwent mTOR inhibitor treatment, there were significant differences between wild-type p53 and missense mutant p53 cells (**Figure 6G-6L, Supplementary Figure 5G-5J**). The distribution of mutant p53 determines the efficiency of PD-L1 transformed into clinical benefits for colorectal cancer.

The translation suppression protein of 4EBP1 could bind to eIF4E to limit the translational process. Phosphorylation and activation of 4EBP1 at serine and threonine residues could initiate translational initiation, which makes it release from eIF4E to form a complex at the 5' end of mRNAs 30. Further GSEA analysis of relative mRNA expression revealed that p53 and eIF4E highly correlated with PD-L1 (**Figure 7A-7C**). We showed that translational upregulation of PD-L1 was effectively targeted by phosphorylation of 4EBP1 to escape from the immune response (**Figure 7D, 7E, Supplementary Figure 6A, 6B**). Moreover, the inhibitors of PD-1/PD-L1 inhibitor 1 and BMS-1001 effectively inhibited PD-L1 (**Figure 7F, 7G, Supplementary Figure 6C, 6D, 7C, 7D**). Since our data showed PHLPP2 activity correlated with PD-L1 expression in mutant p53-driven cancer cells, the activation/inhibition of PHLPP2 or eIF4E/4EBP1 might be some of the factors that influence response to ICIs, and could be therefore a future direction for research.

Based on our mechanistic studies, we demonstrated that mutant p53 inhibited the expression of PHLPP2 and activated AKT, which promoted tumor progression. Next, our research showed that p53 mutation and wild-type were significantly different in terms of associations with PD-L1 and tumor microenvironment (TME) composition. Besides, mutant p53 indicated low survival through the 4EBP1-eIF4E translation axis. Collectively, we uncover a new mechanism that the missense mutant p53 has Gain-of-Function to exhibit high PD-L1. Thus, the cooperation between 4EBP1 and eIF4E dependent translation has emerged as a link by p53 mutation promoting tumor progression and PD-L1 dynamics via empowering the PHLPP2-AKT based pathway.

## Supplementary Material

Supplementary figures, tables, and code.

## Figures and Tables

**Table 1:** Summary details of some gene expression in colorectal cancer tissues by High-throughput analysis

Characteristic	P1	P2	P3	P4	P5	P6	P7	P8	P9	P10	P11	P12	P13
Age	59	52	39	59	44	41	48	61	47	55	65	74	67
Gender	Male	Female	Female	Male	Female	Male	Male	Male	Male	Male	Male	Male	Male
Pathology	Colon cancer	Rectal cancer	Rectal cancer	Colon cancer	intestinal sarcoma	Colon cancer	Colon cancer	Colon cancer	Colon cancer	Colon cancer	Colon cancer	Colon cancer	Colon cancer
TP53	V173L	WT	G266*P250L	WT	WT	WT	R175H	R282W	R1114*S1465Wfs*3A386T	WT	WT	R175H	V274F
KRAS	K117N,R149G	WT	G12S	WT	WT	WT	WT	G12D	WT	G12D	G12C	WT	G12S
APC	R216X,K1250X	WT	WT	WT	WT	WT	WT	K1308* H298Pfs*7	WT	R876* R1450*	T1556Nfs*3	E1397*	S1407*
PIK3CA	C901F	WT	WT	WT	WT	WT	WT	WT	WT	R88Q	E545G H1047R	WT	WT
AKT3	WT	WT	WT	WT	WT	WT	WT	WT	WT	WT	R66L	WT	WT
EGFR	WT	WT	WT	WT	WT	WT	WT	WT	R451C	WT	WT	WT	WT
BRAF	WT	WT	WT	WT	WT	WT	WT	WT	WT	WT	WT	WT	WT
Immunotherapy	Yes	No	No	No	No	No	No	No	Yes	No	No	No	No

**Table 2:** Summary described more detail of some primers of the PCR and ChIP assay

Primer pair	Sequence (5'-3')
PHLPP2-Forward Primer1	TGGAACCTACTGAACGACCTC
PHLPP2-Reverse Primer1	ATCCAAACGATCCATGTGGCA
PD-L1-Forward Primer	TGGCATTTGCTGAACGCATTT
PD-L1-Reverse Primer	TGCAGCCAGGTCTAATTGTTTT
Human-β-Actin-Forward Primer	TGGCACCCAGCACAATGAA
Human-β-Actin-Reverse Primer	CTAAGTCATAGTCCGCCTAGAAGCA
GAPDH-Forward Primer	TACTAGCGGTTTTACGGGCG
GAPDH-Reverse Primer	TCGAACAGGAGGAGCAGAGAGCGA
PHLPP2-1-Forward Primer	CACTTGGCTTATTTGGGATG
PHLPP2-1-Reverse Primer	CTCTCCTGTTGACAGCATTC
PHLPP2-2-Forward Primer	GAATGCTGTCAACAGGAGA
PHLPP2-2-Reverse Primer	ACAATACTTATGTTTCACCCTG
PHLPP2-3-Forward Primer	ACAGGGTGAAACATAAGTATTG
PHLPP2-3-Reverse Primer	GGTAGGAGAATCACTTGAAC
PHLPP2-4-Forward Primer	CTTCACACCTCTGCCTCCCAG
PHLPP2-4-Reverse Primer	CAGCACTTTGGGAGGCCAAG

## Abbreviations

PD-L1: programmed death-ligand 1
PI3K: phosphoinositide 3-kinase
AKT: protein kinase B
mTOR: the mammalian target of rapamycin
eIF4E: eukaryotic translation initiation factor 4 epsilon
PFTα: Pifithrin-α HBr
DCs: dendritic cells
PDB: the Protein Data Bank
TME: tumor microenvironment
TAMs: Tumor-associated macrophages
TCGA: the Cancer Genome Atlas
TCPA: the Cancer Proteome Atlas
