# Supplementary material for: Mutant p53 achieved Gain-of-Function by promoting tumor growth and immune escape through PHLPP2/AKT/PD-L1 pathway
Source: Int J Biol Sci. 2022 Mar 14;18(6):2419–38. doi: 10.7150/ijbs.67200 (PMC8990467; doi:10.7150/ijbs.67200)
Supplement: Supplementary file 1 — Supplementary figures, tables, and code. [file ijbsv18p2419s1.pdf]

Supplementary Figure 1

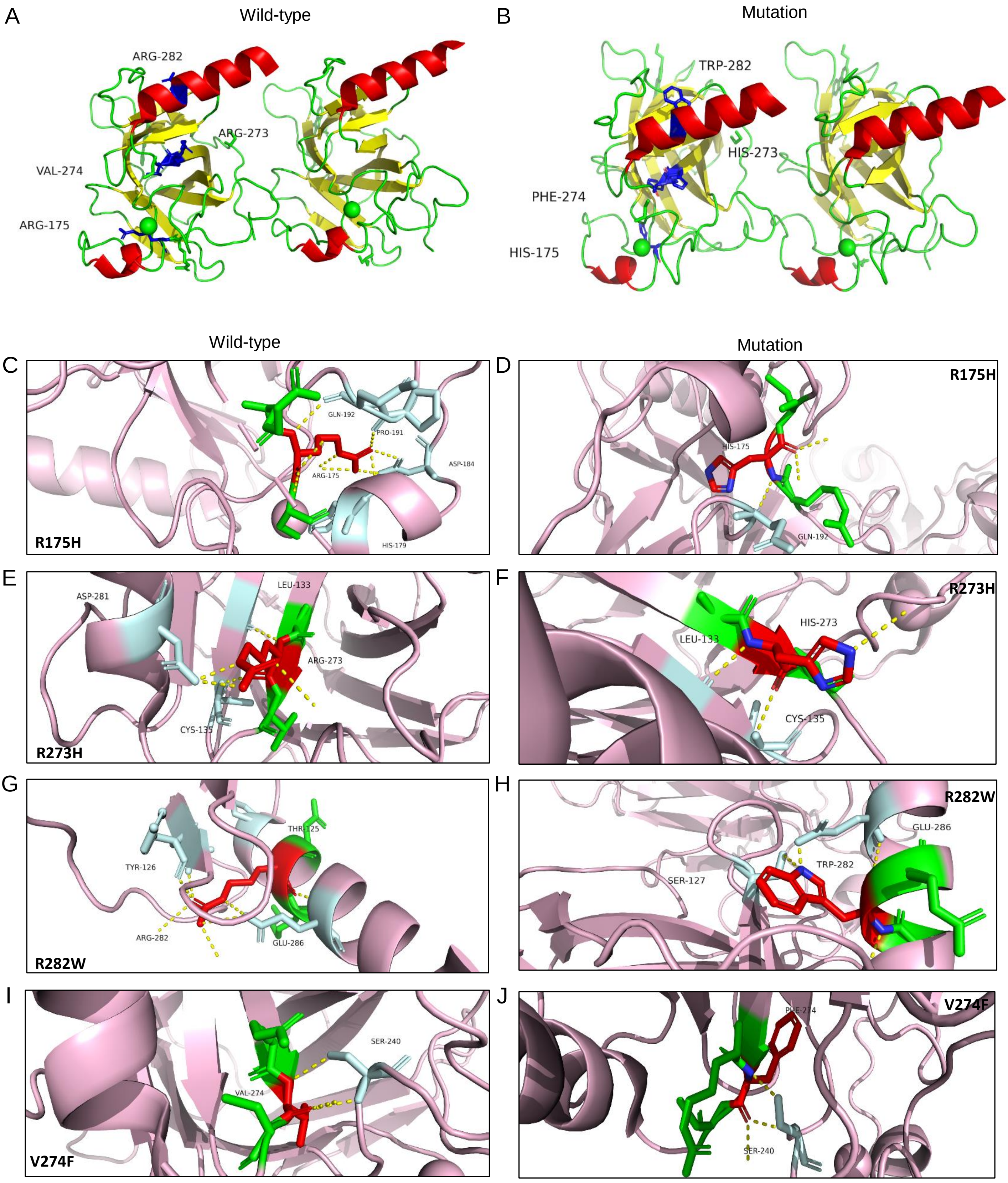

Supplementary Figure 2

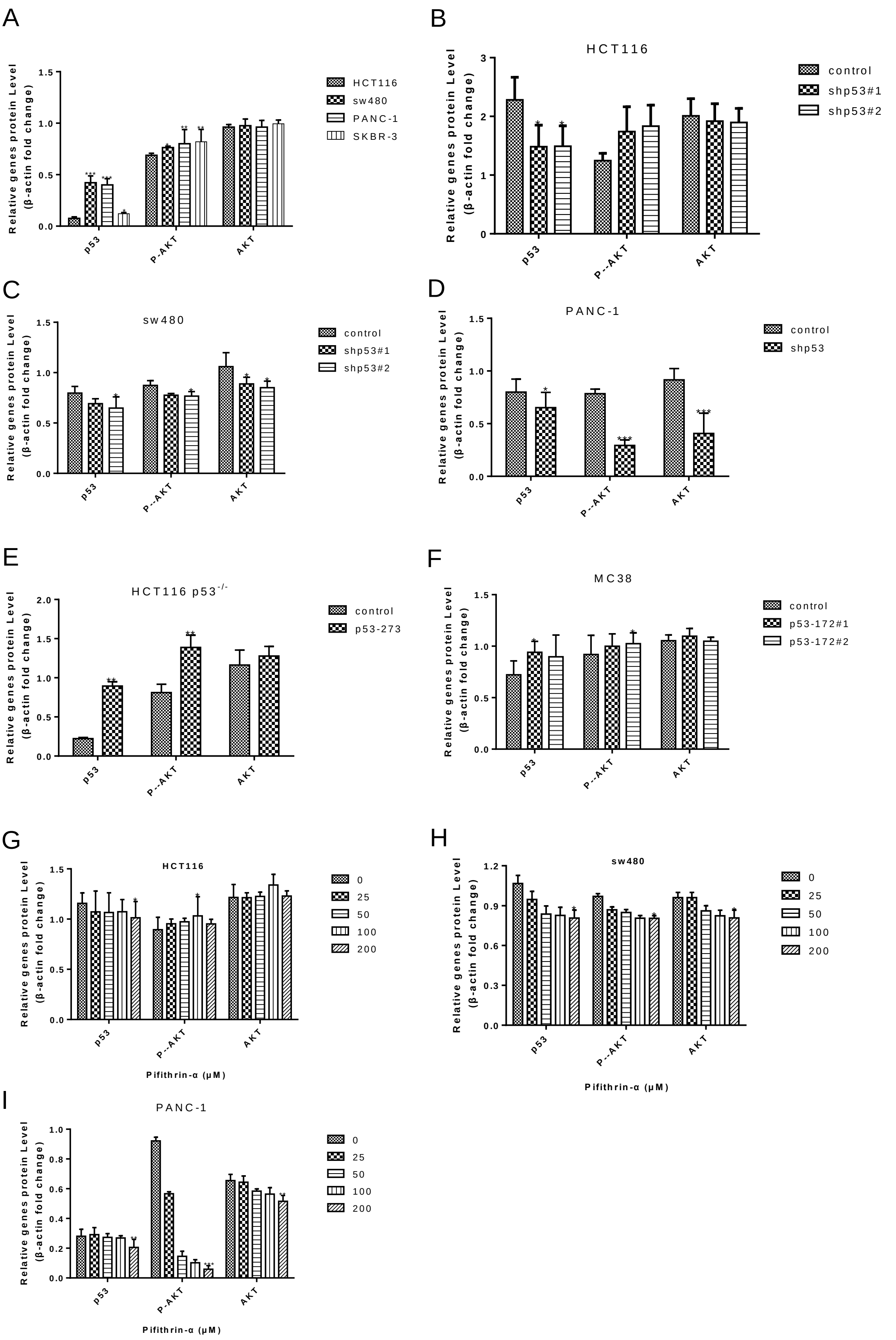

Supplementary Figure 3

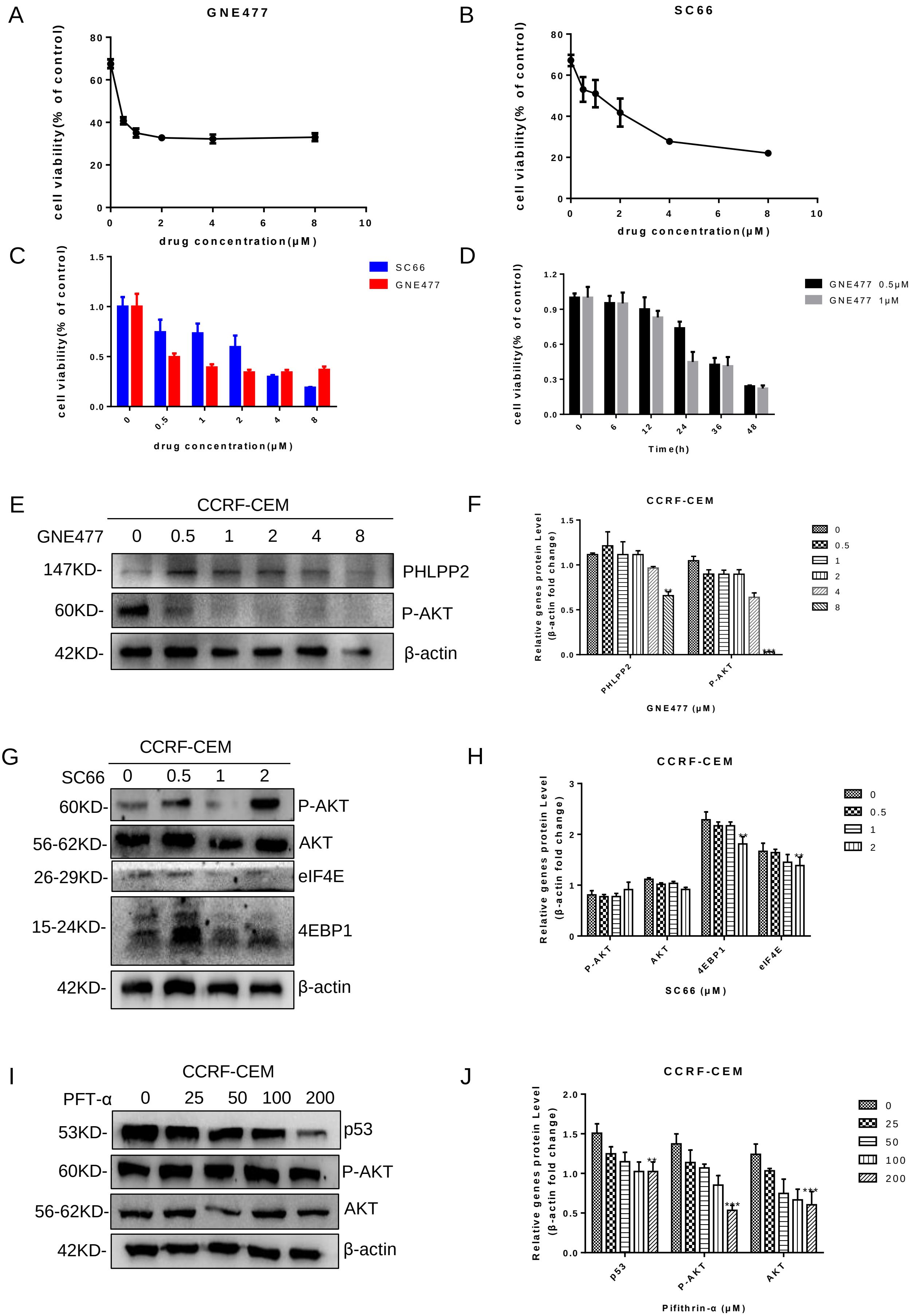

Supplementary Figure 4

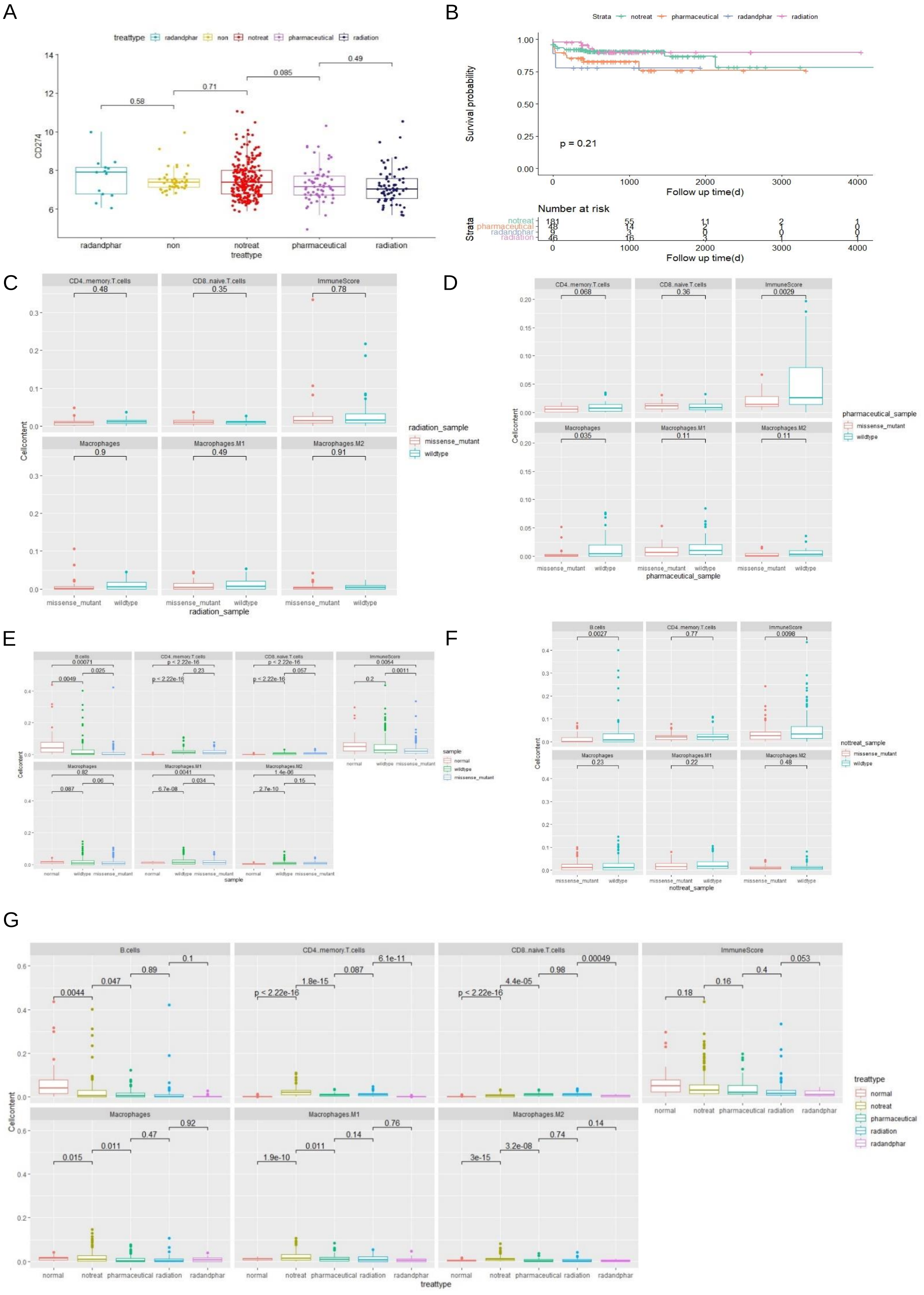

Supplementary Figure 5

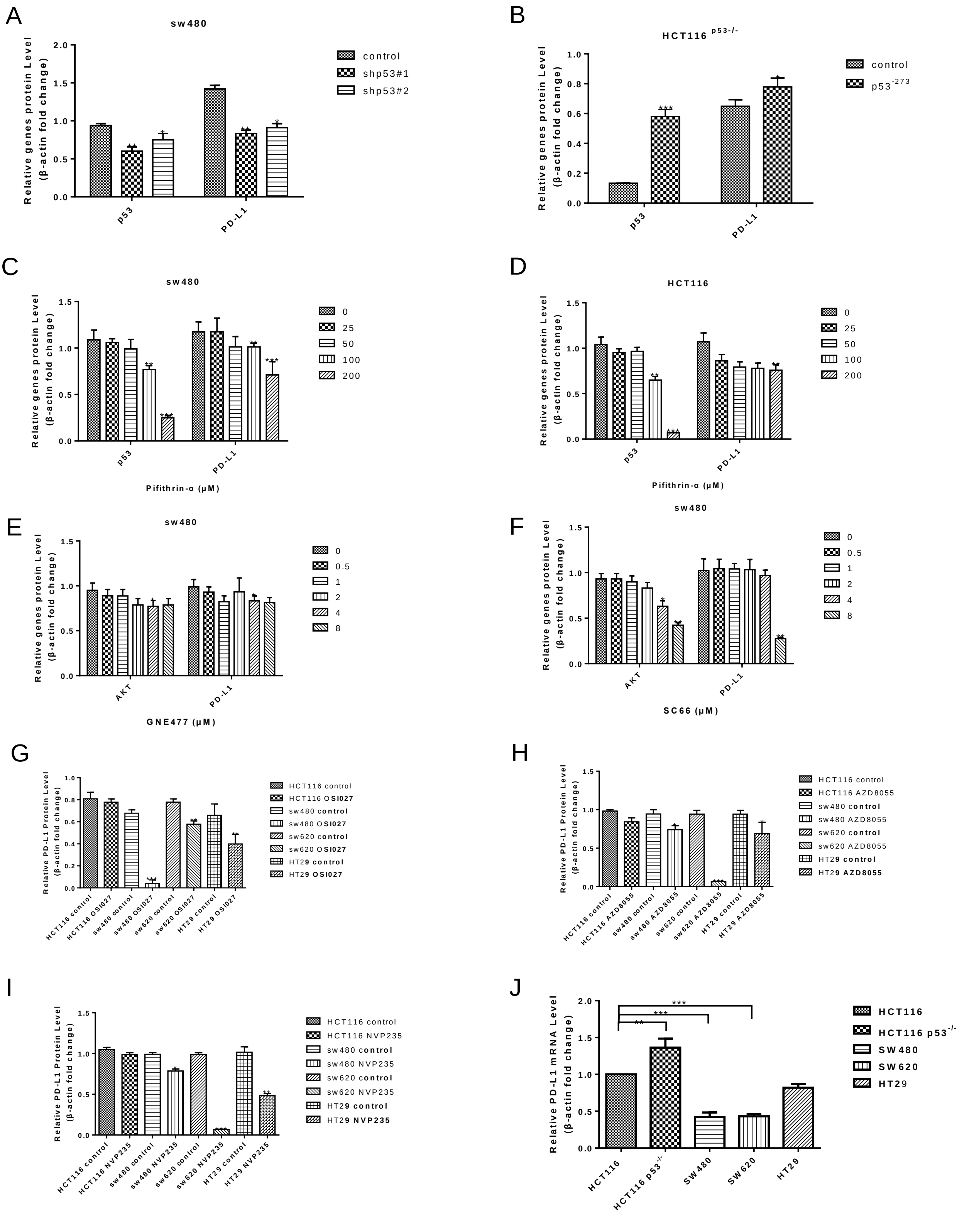

Supplementary Figure 6

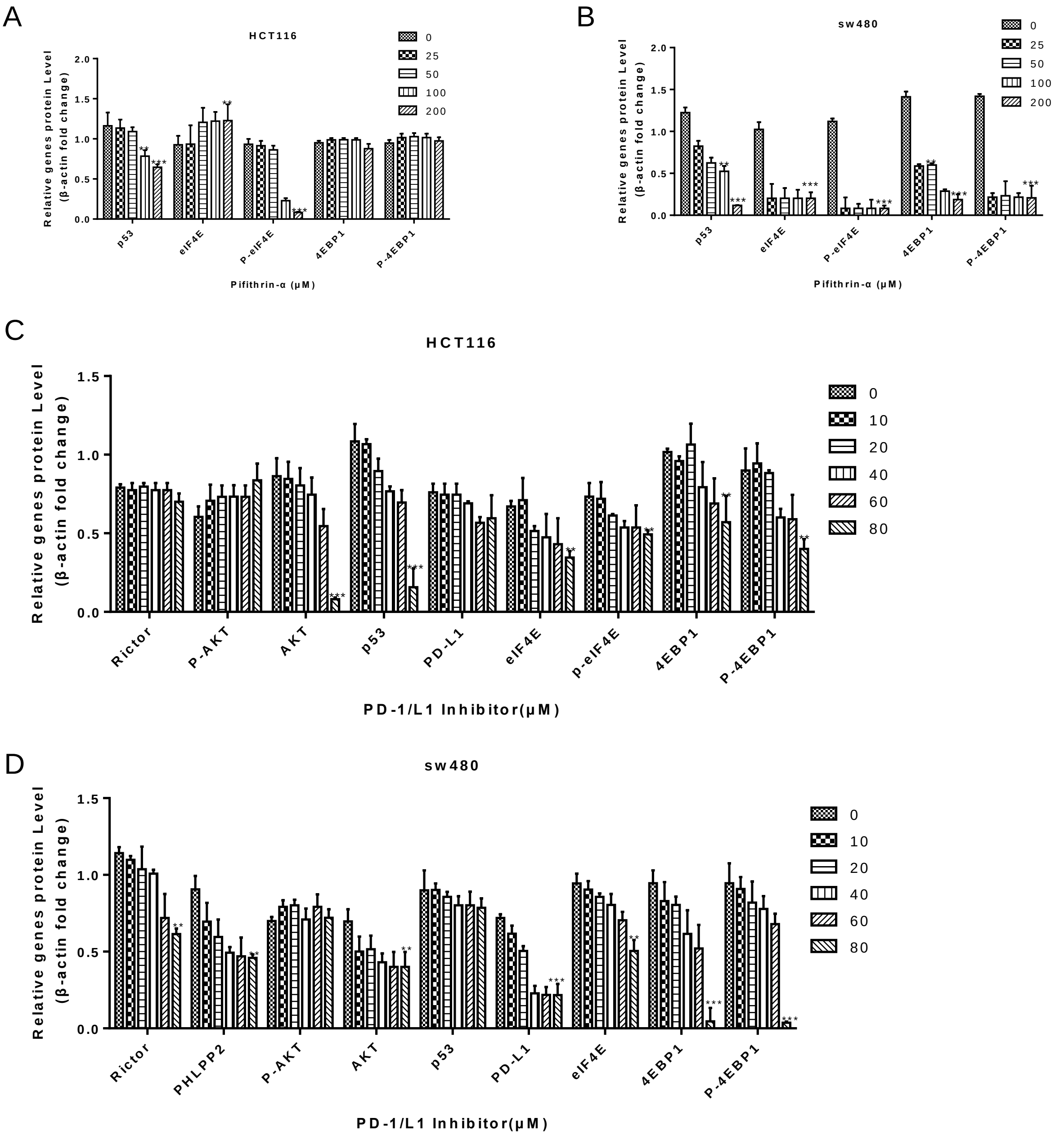

Supplementary Figure 7

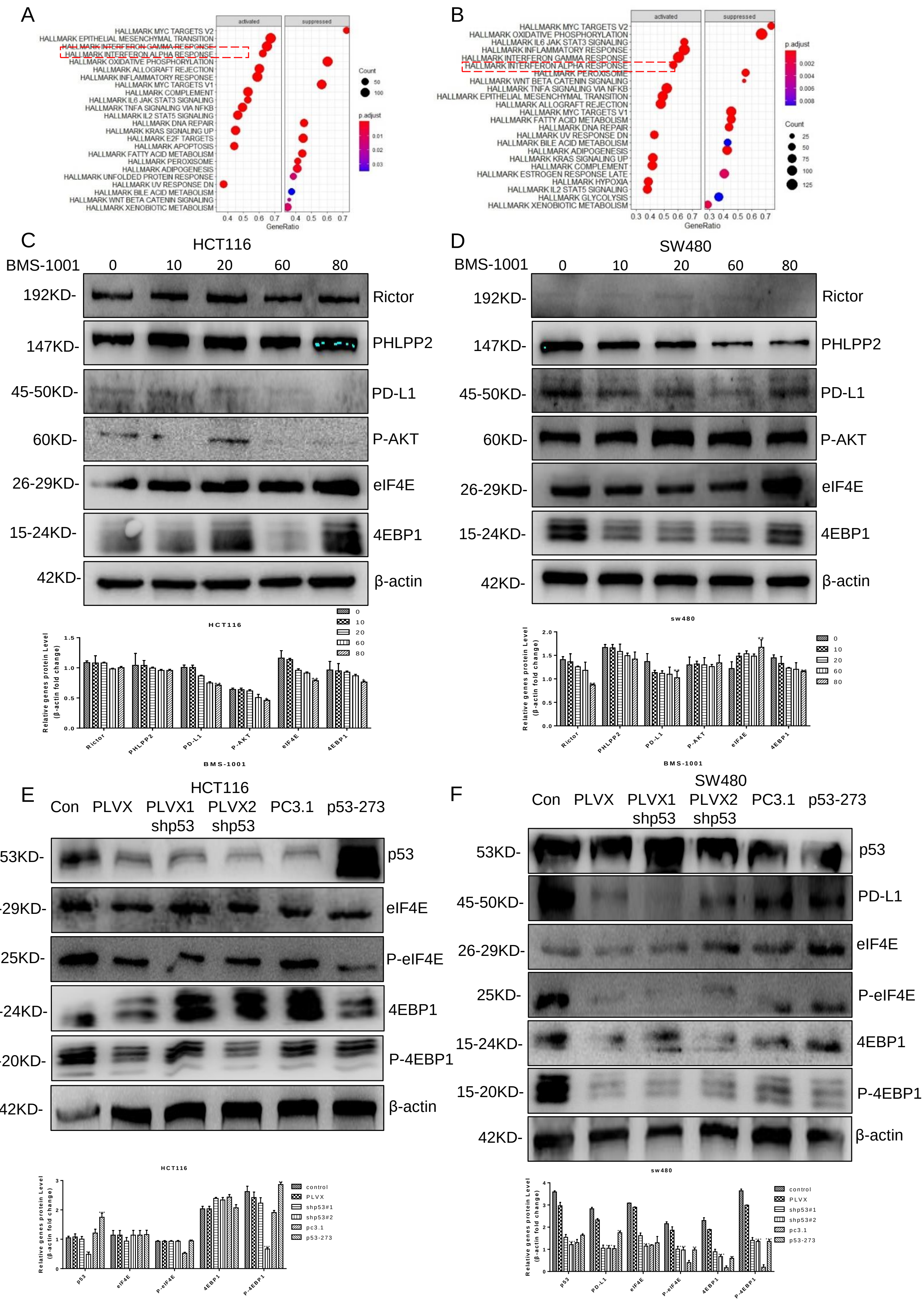

Table 1. Summary details of some gene expression in colorectal cancer tissues by High-throughput analysis.

Characteristic	P1	P2	P3	P4	P5	P6	P7	P8	P9	P10	P11	P12	P13
Age	59	52	39	59	44	41	48	61	47	55	65	74	67
Gender	Male	Female	Female	Male	Female	Male	Male	Male	Male	Male	Male	Male	Male
Pathology	Colon cancer	Rectal cancer	Rectal cancer	Colon cancer	intestinal sarcoma	Colon cancer	Colon cancer	Colon cancer	Colon cancer	Colon cancer	Colon cancer	Colon cancer	Colon cancer
TP53	V173L	WT	G266* P250L	WT	WT	WT	R175 H	R282W	R1114* S1465 Wfs*3 A386T	WT	WT	R175H	V274F
KRAS	K117N, R149G	WT	G12S	WT	WT	WT	WT	G12D	WT	G12D	G12C	WT	G12S
APC	R216X, K1250 X	WT	WT	WT	WT	WT	WT	K1308* H298Pfs*7	WT	R876* R1450 *	T1556N fs*3	E1397*	S1407*
PIK3CA	C901F	WT	WT	WT	WT	WT	WT	WT	WT	R88Q	E545G H1047R	WT	WT
AKT3	WT	WT	WT	WT	WT	WT	WT	WT	WT	WT	R66L	WT	WT
EGFR	WT	WT	WT	WT	WT	WT	WT	WT	R451C	WT	WT	WT	WT
BRAF	WT	WT	WT	WT	WT	WT	WT	WT	WT	WT	WT	WT	WT
Immunotherapy	Yes	No	No	No	No	No	No	No	Yes	No	No	No	No

Table 2. Summary described more detail of some primers of the PCR and ChIP assay.

Primer pair	Sequence (5’-3’)
PHLPP2-Forward Primer1	TGGAACCTACTGAACGACCTC
PHLPP2-Reverse Primer1	ATCCAAACGATCCATGTGGCA
PD-L1-Forward Primer	TGGCATTTGCTGAACGCATTT
PD-L1-Reverse Primer	TGCAGCCAGGTCTAATTGTTTT
Human-β-Actin-Forward Primer	TGGCACCCAGCACAATGAA
Human-β-Actin-Reverse Primer	CTAAGTCATAGTCCGCCTAGAAGCA
GAPDH-Forward Primer	TACTAGCGGTTTTACGGGCG
GAPDH-Reverse Primer	TCGAACAGGAGGAGCAGAGAGCGA
PHLPP2-1-Forward Primer	CACTTGGCTTATTTGGGATG
PHLPP2-1-Reverse Primer	CTCTCCTGTTGACAGCATTC
PHLPP2-2-Forward Primer	GAATGCTGTCAACAGGAGA
PHLPP2-2-Reverse Primer	ACAATACTTATGTTTCACCCTG
PHLPP2-3-Forward Primer	ACAGGGTGAAACATAAGTATTG
PHLPP2-3-Reverse Primer	GGTAGGAGAATCACTTGAAC
PHLPP2-4-Forward Primer	CTTCACACCTCTGCCTCCCAG
PHLPP2-4-Reverse Primer	CAGCACTTTGGGAGGCCAAG

Code

```
setwd('D:/rwork/rdata/mut-p53')
load(file = "mRNA_exprSet.Rdata")
save(clin,file = "clin.Rdata")
View(res)
a <- as.numeric(rownames(clin.miss.mutwild))
expert.mut <- mRNA_exprSet[,c(1,a+1)]
clin.mut<- clin.miss.mutwild[,-2]
clin.mut$sample <- as.factor(clin.mut$sample)
#DEseq2
setwd('D:/rwork/rdata/mut-p53')
library(DESeq2)
mycounts <- expert.mut
dds <-DESeqDataSetFromMatrix(countData=mycounts,
                             colData=clin.mut,
                             design=~sample,
                             tidy=TRUE)
dds <- dds[rowSums(counts(dds))>0,]

vsd <- vst(dds, blind = FALSE)
vst <- as.data.frame(assay(vsd))
save(vst,file = "mrna.vst.Rdata")
load(file = "colon.protein.expr.Rdata")
dds <- DESeq(dds)
save(dds,file = "dds.mrna.Rdata")
res <- results(dds, tidy=TRUE)
save(res,file = "res.mrna.Rdata")
q <- which(res$pvalue<0.05&res$log2FoldChange< -1)
w <- which(res$pvalue<0.05&res$log2FoldChange> 1)
lnc.diff<- res[c(q,w),]
lncrna <- lnc.diff %>% tidyr::separate(row,into = c("gene_id"),sep="\\ ")
save(lncrna,file = "lnrna.Rdata")
View(mRNA.expr.vst)

library(ggstatsplot)
library(ggplot2)
vst.test <- as.data.frame(t(vst))
ggscatterstats(data = colon.protein.expr,
               x = "p53",
               y = "PD-L1",ylab = "PD-L1",
               xlab = "p53",
               centrality.para = "mean",
               xfill = "#CC79A7",
```

```
                  yfill = "#009E73",
                  marginal.type = "histogram",
                  title = "Relationship between PD-L1 and p53")

mRNA.expr.vst = data.frame(sample = "",vst.test[,c(9252,13886,15504)])
mRNA.expr.vst$sample <- clin.mut$sample
library(ggpubr)
mRNA.expr.vst$sample <- factor(mRNA.expr.vst$sample,levels = c("Wildtype","Missense_Mutation"),ordered = F)
my_comparisons <- list(
  c("Wildtype", "Missense_Mutation")
)
ggboxplot(
  mRNA.expr.vst, x = "sample", y = "AKT1",ylab = "AKT mRNA expression",
  color = "sample",palette = c(Missense_Mutation="#E7B800",Wildtype="#00AFBB"),
  add = "jitter"
)+
  stat_compare_means(comparisons = my_comparisons, method = "t.test")

colon.clin <- data.table::fread("E:/guge/clinical.cart.2020-12-02/clinical.tsv",data.table = F)
View(colon.protein.expr)
colon.protein.expr <- data.table::fread("E:/guge/TCGA-COAD-L3-S42/tmp/TCGA-COAD-L3-S42.csv",data.table = F)
View(colon.protein.expr)
colon.protein.expr$Cancer_Type <- substr(colon.protein.expr$Sample_ID,start = 1,stop = 12)
colnames(colon.protein.expr)[2] <- "clin"
colnames(colon.protein.expr)[3] <- "sample"
clin.mutandwild$sample <- as.character(clin.mutandwild$sample)
colon.protein.expr$sample <- ""
for (i in 1:319) {
  for(j in 1:354){
    if(colon.protein.expr$clin[i]==clin.miss.mutwild$clinshort[j]){
      colon.protein.expr$sample[i] <- clin.miss.mutwild$sample[j]
    }
  }
}
x <- which(colon.protein.expr$sample=="")
colon.protein.expr <- colon.protein.expr[-c(x),]
a <- as.data.frame(colnames(colon.protein.expr))
```

```
protein.expr = data.frame(colon.protein.expr[,c(3,92,226)])
library(ggpubr)
colon.protein.expr$sample <- factor(colon.protein.expr$sample,levels = c("Wildtype","Missense_Mutation"),ordered = F)
my_comparisons <- list(
  c("Wildtype", "Missense_Mutation")
)
ggboxplot(
  colon.protein.expr, x = "sample", y = "Akt_pT308",ylab = "Akt_pT308 protein expression",
  color = "sample",palette = c(Missense_Mutation="#E7B800",Wildtype="#00AFBB"),
  add = "jitter"
)+
  stat_compare_means(comparisons = my_comparisons,method = "wilcox.test")
method = "wilcox.test"
save(colon.protein.expr,file = "colon.protein.expr.Rdata")

clin.mutandwild <- data.frame(clin.mutandwild,days="",status="")
for (i in 1:427) {
  for (j in 1:473) {
    if(clin$case_submitter_id[i]==clin.mutandwild$clinshort[j]){
      clin.mutandwild$days[j] <- clin$days_to_last_follow_up[i]
      clin.mutandwild$status[j] <- clin$vital_status[i]
    }
  }
}

x <- which(clin.mutandwild$days=="'--")
clin.mutandwild <- clin.mutandwild[-c(x),]

library(survminer)
library(survival)
clin.mutandwild$status <- as.numeric(clin.mutandwild$status)
clin.mutandwild$days <- as.numeric(clin.mutandwild$days)
save(clin.survival,file = "clin.survival.Rdata")
load(file = "clin.survival.Rdata")
for(i in 1:397){
  if(clin.survival.mutp53$status[i]==2){
    clin.survival.mutp53$status[i] <- 1
  }
}
fit <- surv_fit(Surv(days, status) ~ sample, data = clin.survival.mutp53)
surv_summary(fit, data = clin.mutandwild)
```

```
survdiff(Surv(days, status) ~ sample, data = clin.mutandwild)
survdiff(formula = Surv(days, status) ~ sample, data = clin.mutandwild)
ggsurvplot(fit, data = clin.survival.mutp53, linetype = c('solid', 'solid'), surv.median.line = 'none',
           pval = T, risk.table = T, palette = 'Set2', legend.labs = c("Missense_","wildtype"),xlab = "Follow up time(d)",ylab = "Survival probability")

clin.survival.mutp53 <- clin.mutandwild
save(clin.survival.mutp53,file = "clin.survival.mutp53.Rdata")
load(file = "clin.survival.mutp53.Rdata")

View(clin.273)
for (i in 1:397) {
  for (j in 1:427) {
    if(clin.survival.mutp53$clinshort[i]==clin$case_submitter_id[j]){
      clin.survival.mutp53$t[i] <- clin$ajcc_pathologic_t[j]
      clin.survival.mutp53$n[i] <- clin$ajcc_pathologic_n[j]
      clin.survival.mutp53$m[i] <- clin$ajcc_pathologic_m[j]
    }
  }
}
x <- which(clin.survival.mutp53$t=="T1")
clin.t1 <- clin.survival.mutp53[c(x),]
fit <- surv_fit(Surv(days, status) ~ sample, data = clin.t1)
ggsurvplot(fit, data = clin.t1, linetype = c('solid', 'solid'), surv.median.line = 'none',
           pval = T, risk.table = T, palette = 'Set2', legend.labs = c("Missense_mutation","Wildtype"),xlab = "Follow up time(d)",ylab = "Survival probability")

setwd('D:/rwork/rdata/p53-273')
load(file = "clin.273.Rdata")
for(i in 1:427){
  if(clin$vital_status[i]==2){
    clin$vital_status[i] <- 0
  }
}
for (i in 1:271) {
  for (j in 1:427) {
    if(clin.273$clinshort[i]==clin$case_submitter_id[j]){
      clin.273$days[i] <- clin$days_to_last_follow_up[i]
      clin.273$status[i] <- clin$vital_status[i]
    }
  }
}
```

```
clin.273$status <- as.numeric(clin.273$status)
clin.273$days <- as.numeric(clin.273$days)
fit <- surv_fit(Surv(days, status) ~ sample, data = clin.273)
ggsurvplot(fit, data = clin.273, linetype = c('solid', 'solid'), surv.median.line = 'none',
           pval = T, risk.table = T, palette = 'Set2', legend.labs =
c("Missense_mutation","Wildtype"),xlab = "Follow up time(d)",ylab = "Survival
probability")

setwd('D:/rwork/rdata/p53-273-wildtype')
load(file = "clin.mRNA.Rdata")
View(clin.mRNA)
for (i in 1:52) {
  for (j in 1:427) {
    if(clin.mRNA$clinshort[i]==clin$case_submitter_id[j]){
      clin.mRNA$days[i] <- clin$days_to_last_follow_up[i]
      clin.mRNA$status[i] <- clin$vital_status[i]
    }
  }
}

clin.miss1$status <- as.numeric(clin.miss1$status)
clin.miss1$days <- as.numeric(clin.miss1$days_to_last_follow_up)
fit <- surv_fit(Surv(status, days) ~ sample, data = clin.miss1)
ggsurvplot(fit, data = clin.miss1, linetype = c('solid', 'solid'), surv.median.line = 'none',
           pval = T, risk.table = T, palette = 'Set2', legend.labs =
c("Missense_mutation","Wildtype"),xlab = "Follow up time(d)",ylab = "Survival
probability")


x <- which(clin.miss1$days_to_last_follow_up=="'--")
y <- which(clin$sample=="Missense_Mutation")
clin.miss1 <- clin.miss1[,-c(2,3)]
clin.miss1 <- data.frame()
clin.miss1 <- clin.miss[,c(2,19,51)]
View(index)
setwd('D:/rwork/rdata/mut-p53')
save(clin.miss1,file = "clin.miss1.Rdata")
load(file = "clin.miss1.Rdata")
for(i in 1:325){
  if(clin.miss1$status[i]==2){
    clin.miss1$status[i] <- 1
  }
}
```

```
colon <- data.table::fread("E:/edge 下 载 /gdc_download_20211116_090632.397174/maf/TCGA.COAD.mutect.somatic.maf/TCGA.COAD.mutect.somatic.maf",data.table = F)
colon.clin <- data.table::fread("E:/edge 下 载 /clinical.cart.2021-11-16/clinical.tsv",data.table = F)

colon.maf <- colon
colon.maf$Tumor_Sample_Barcode <- substr(colon$Tumor_Sample_Barcode,start=1,stop=12)
colnames(colon.clin)[2] <- "Tumor_Sample_Barcode"
x <- which(colon.clin$vital_status=="Dead")
colon.clin$vital_status[x] <- 0
colon.clin$days_to_last_follow_up <- as.numeric(colon.clin$days_to_last_follow_up)
colon1 = read.maf(maf=colon.maf,clinicalData = colon.clin)
mafSurvival(maf=colon1,
            genes = 'TP53',
            time = 'days_to_last_follow_up',
            Status = 'vital_status',
            isTCGA = TRUE)
prog_geneset = survGroup(maf = colon1,
                         top = 20,
                         geneSetSize = 1,
                         time = 'days_to_last_follow_up',
                         Status = 'vital_status',
                         verbose = FALSE)

index <- duplicated(colon.clin[,2])
a <- colon.clin[!index,]
View(clin.273)
a <- data.frame(id=a$Tumor_Sample_Barcode,sample="",status=a$vital_status,
                days=a$days_to_last_follow_up,stringsAsFactors = F)

x <- which(colon.tp53$Variant_Classification=="Missense_Mutation")
colon.tp53 <- colon.maf[c(x),]
colon.miss <- colon.tp53[c(x),]
y <- as.data.frame(table(colon.maf$Tumor_Sample_Barcode))
for (i in 1:399) {
  for (j in 1:447) {
    if(y$Var1[i]==a$id[j]){
      a$sample[j] <- "mutWildtype"
    }
  }
}
for (i in 1:229) {
```

```
    for (j in 1:447) {
        if(colon.tp53$Tumor_Sample_Barcode[i]==a$id[j]){
            a$sample[j] <- "mutTP53"
        }
    }
}
for (i in 1:162) {
    for (j in 1:447) {
        if(colon.miss$Tumor_Sample_Barcode[i]==a$id[j]){
            a$sample[j] <- "Missense_Mutation"
        }
    }
}

x <- which(a$sample=="mutTP53")
a$sample[c(x)] <- "Wildtype"
clin.new <-a
setwd('D:/rwork/rdata/mut-p53')
save(clin.new,file = "clin.new.Rdata")
x <- which(b$sample=="175mut")
b$sample[c(x)] <- "Mutation"
b <- b[-c(x),]
a$status <- as.numeric(a$status)
a$days <- as.numeric(a$days)
b$sample <- factor(b$sample,levels = c("Wildtype","Mutation"),ordered = F)
fit <- surv_fit(Surv( days,status) ~ sample, data = b)
ggsurvplot(fit, data = b, linetype = c('solid', 'solid'), surv.median.line = 'none',
           pval = T, risk.table = T, palette = 'Set2', legend.labs =
c("Wildtype","Mutation"),xlab = "Follow up time(d)",ylab = "Survival probability")
View(clin.175)
for (i in 1:79) {
    for (j in 1:447) {
        if(b$id[i]==a$id[j]){
            a$sample[j] <- b$sample[i]
        }
    }
}

setwd('D:/rwork/rdata/mut-p53')
load(file = "xcell.Rdata")
x <- which(clin.175$sample=="175mut")
x <- which(b$sample=="248mut")
b <- a[-c(x),]
c <- clin.175[c(x),]
```

```
b <- b[-c(x),]
for (i in 1:27) {
    for (j in 1:447) {
        if(c$clinshort[i]==a$id[j]){
            a$sample[j] <- c$sample[i]
        }
    }
}

setwd('D:/rwork/rdata')
load(file = "mRNA_exprSet.Rdata")
View(clin.miss.mutwild)
a <- clin.new
x <- which(a$sample=="mutTP53")
a <- a[-c(x),]
x <- colnames(b)
c <- mRNA_exprSet
rownames(b) <- b$gene_id
b <- b[,-1]
x <- data.frame(clin = colnames(b),clinshort = substr(colnames(b),start = 1,stop = 12),sample="")
y <- which(substring(x$clin,14,15)=="11")
x <- x[-c(y),]
for (i in 1:331) {
    for (j in 1:473) {
        if(a$id[i]==x$clinshort[j]){
            x$sample[j] <- a$sample[i]
        }
    }
}
y <- which(x$sample=="")
x <- x[-c(y),]
y <- as.numeric(rownames(x))
b <- mRNA_exprSet[,-1]
c <- c[,c(y)]
rownames(b) <- mRNA_exprSet$gene_id
xCell    = xCellAnalysis(c, rnaseq = TRUE)
y <- rownames(b)
write.csv(y,file = "y.csv")
library(xlsx)
y <- read.xlsx("y.xlsx",row.names = FALSE)
y <- y[,-1]
rownames(b) <- y
z <- read.xlsx("z.xlsx",sheetIndex = 1)
```

```
z <- z[,-1]
View(xcell)
c <- c[1:19566,]
c$gene_id <- z
rownames(b) <- b$gene_id
c <- b[,-1]
library(clusterProfiler)
gene = bitr(z, fromType="SYMBOL", toType="ENTREZID",
OrgDb="org.Hs.eg.db")

b <- dplyr::distinct(c,gene_id,.keep_all=TRUE)
save(xcell,file = "xcell.Rdata")
save(clin.miss.mutwild,file = "clin.miss.mutwild.Rdata")
clin.miss.mutwild <- x
load(file = "clin.miss.mutwild.Rdata")

xcell <- data.frame(t(xCell))
xcell$sample <- clin.miss.mutwild$sample
x <- which(clin.miss.mutwild$sample=="mutWildtype")
clin.miss.mutwild$sample[c(x)] <- "Wildtype"
library(ggpubr)
expr_gather$sample <- factor(expr_gather$sample,levels =
c("Missense_Mutation","Wildtype"),ordered = F)
my_comparisons <- list(
  c("Wildtype", "Missense_Mutation")
)
ggboxplot(
  expr_gather, x = "Cellnames", y = "Cellcontent",ylab = "Cellcontent",
  color = "sample",palette =
c(Missense_Mutation="#E7B800",Wildtype="#00AFBB"),
  add = "jitter"
)+
  stat_compare_means(comparisons = my_comparisons, method = "t.test")


index <- which(colnames(xcell) %in%
c("CD4..memory.T.cells","CD8..naive.T.cells","Macrophages","Macrophages.M1","
Macrophages.M2","ImmuneScore"))
expr8 <- xcell[c(index,68)]
expr_gather <- tidyr::gather(expr8,key = Cellnames, value = Cellcontent,-sample)
ggplot(
  expr_gather,
  aes(x = sample, y = Cellcontent,color=sample)
) +
```

```
  geom_boxplot()+
  facet_wrap(~Cellnames,nrow = 2)+
  stat_compare_means(comparisons = my_comparisons, method = "t.test")

load(file = "xcell.Rdata")
View(expr_gather)
bk <- c(seq(-1,-0.1,by=0.01),seq(0,6,by=0.01))
library(pheatmap)
pheatmap(heatdata1,
         cluster_rows = FALSE,
         cluster_cols = TRUE,
         annotation_col =coldata1,
         show_colnames = F,
         annotation_legend=TRUE,
         scale = "row",
         ##color =colorRampPalette(c("blue", "white","red"))(100),
         fontsize = 10,cellwidth = 1.5,cellheight = 18,
         main = "TCGA immunecells heatmap",
        color = c(colorRampPalette(colors = c("white","red"))(length(bk)/2),colorRampPalette(colors = c("red","black"))(length(bk)/2)),
        legend_breaks=seq(-8,8,2),
         breaks=bk
)

x <- colnames(xcell)
heatdata <- xcell[,c(4,6,7,11,12,32,33,34,38,46,61,62,63,64,65,66,67)]
heatdata <- data.frame(t(heatdata))
coldata1 <- data.frame(sample=c(1:354))
for (i in 1:354) {
  coldata1$sample[i] <- coldata$sample[i]
  rownames(coldata1)[i] <- coldata$clin[i]
}
colnames(heatdata) <- rownames(xcell)

x <- which(coldata$sample=="Wildtype")
y <- which(coldata$sample=="Missense_Mutation")
coldata1 <- coldata[c(x,y),]
heatdata1 <- heatdata[,c(x,y)]
coldata <- clin.miss.mutwild[c(x,y),]
rownames(coldata1) <- coldata$clin
save(coldata1,file = "coldata1.Rdata")
load(file = "coldata1.Rdata")
coldata1$sample <- factor(coldata1$sample,levels =
```

```
c("Wildtype","Missense_Mutation"),ordered = F)
```
